# Enhanced T cell immune activity mediated by Drp1 promotes the efficacy of PD-1 inhibitors in treating lung cancer

**DOI:** 10.1007/s00262-023-03582-5

**Published:** 2024-02-10

**Authors:** Jietao Ma, Jun Song, Xiaofang Yi, Shuling Zhang, Li Sun, Letian Huang, Chengbo Han

**Affiliations:** 1 Department of Oncology, Shengjing Hospital of China Medical University, Shenyang, 110022 China; 2 Department of Oncology, Xiangyang Central Hospital, Affiliated Hospital of Hubei University of Arts and Science, Xiangyang, 441100 China

**Keywords:** Dynamin-related protein 1, T cells, Immunotherapy, Programmed cell death protein 1, Lung cancer

## Abstract

**Background:**

Dynamin-related protein 1 (Drp1)-mediated mitochondrial fission plays important roles in the activation, proliferation, and migration of T cells.

**Methods:**

We investigated the synergistic effect of Drp1-mediated T cell antitumor activities and programmed cell death protein 1 (PD-1) blockade for treating lung cancer through in vitro co-culture experiments and an in vivo nude mouse xenograft model.

**Results:**

High expression levels of Drp1 positively regulated T cell activation, enhanced T cell-induced suppression of lung cancer cells, promoted CD8^+^ T cell infiltration in the tumor and spleen, and significantly enhanced the antitumor immune response of the PD-1 inhibitor pembrolizumab. The mechanism of this synergistic antitumor effect involved the secretion of immune killing-related cytokines and the regulation of the PD-1-ERK/Drp1 pathway in T cells.

**Conclusions:**

Our findings suggest that modifying Drp1 expression in T cells could serve as a potential therapeutic target for enhancing the antitumor immune response in future immunotherapies.

**Supplementary Information:**

The online version contains supplementary material available at 10.1007/s00262-023-03582-5.

## Introduction

The use of immune checkpoint inhibitors targeting programmed cell death protein 1 (PD-1) or programmed death-ligand 1 (PD-L1) has become the standard first-line therapy for advanced non-small cell lung cancer (NSCLC) without actionable oncogenic drivers, with a 5-year survival rate of 10–30%, depending on the PD-L1 expression level. Approximately 10–45% of NSCLC patients show an initial response to immunotherapy; however, the majority of patients experience disease progression within 5 years [1]. The limited efficacy of immunotherapy is partly due to the lack of mutations to tumor-specific antigens, low infiltration of effector T cells, or T cell exhaustion in the tumor microenvironment (TME) [2].

Dynamin-related protein 1 (Drp1) is a highly conserved protein that can dynamically bind to the mitochondrial membrane and drive membrane contraction by utilizing energy from the hydrolysis of guanosine-5ʹ-triphosphate to mediate mitochondrial fission [3]. Drp1-mediated mitochondrial fission enables relocation of the mitochondrial network to different functional regions within the T cell for distinct functions [4]. T cell exhaustion is associated with the impaired function of mitochondrial fission [5]. Under hypoxic conditions, the continuous activation of T cells induces mitochondrial stress, inhibits mitochondrial biogenesis and antioxidant responses, increases the production of reactive oxygen species (ROS), and activates signal transduction pathways, thereby further promoting the transcription of genes related to T cell exhaustion [6]. As mitochondrial fitness (balance between mitochondrial mass and membrane potential) and self-renewal capacity decrease, CD8^+^ T cells gradually accumulate nonfunctional mitochondria and become exhausted [5, 7]. Exhausted T cells are ineffective against tumor cells in anti-PD-1 therapy [8]. The PD-1 signaling pathway enables tumor cells to evade recognition by T cells through immune escape, which can also lead to T cell exhaustion through mitochondrial damage [9, 10].

Mitochondrial dysfunction is an important cause of T cell exhaustion, and Drp1-mediated fission is essential for mitochondrial self-renewal and functional maintenance. Therefore, further elucidation of the role of Drp1-dependent mitochondrial fission in the adaptive immune response will facilitate the development of strategies for treating tumors through the regulation of Drp1 activities. The present study aimed to investigate the effect of Drp1 on the therapeutic efficacy of PD-1/PD-L1 axis blockade in lung cancer both in vitro and in vivo through T-cell transfer therapy.

## Materials and methods

### Study approval

The protocol for blood sample collection was approved by the Human Research Ethics Committee of Shengjing Hospital (Shenyang, China). The protocols of the animal experiments were approved by the Institutional Animal Care and Use Committee of Shengjing Hospital (approval no. 2021PS393K).

### Materials

Peripheral blood samples to isolate CD3^+^ T cells were collected from 10 healthy volunteers aged 18–75 years who had no acute or chronic diseases, particularly diseases involving the immune system, and had no personal or family history of malignancy. A549 lung adenocarcinoma cells were purchased from the Shanghai Institute of Cell Research (Shanghai, China). Female BALB/c nude mice (n = 45; mean body weight, 20 ± 3 g; age, 4 weeks) were obtained from Liaoning Key Laboratory of Research and Application of Animal Models for Environmental and Metabolic Diseases (Shenyang, China).

### Cell infection and validation

**Fig. 1:**
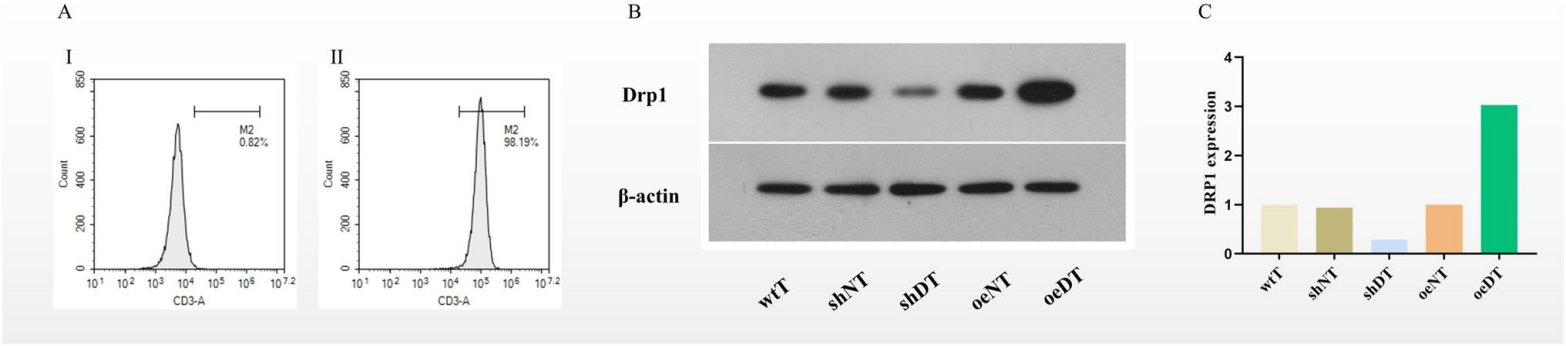
Drp1 protein detected by western blotting assay. **A** CD3^+^ T cells sorted by immunomagnetic beads (I, experimental group; II, control group). **B** Western blotting assay. **C** Western blotting assay grayscale analysis. Abbreviations: wtT: wild-type CD3^+^ T cells; shDT: CD3^+^ T cells with Drp1 knockdown; oeDT: CD3^+^ T cells with Drp1 overexpression; shNT and oeNT: negative control T cells of shDT and oeDT, respectively

Peripheral blood mononuclear cells were isolated from the peripheral blood samples of the healthy volunteers, and CD3^+^ T cells were separated using immunomagnetic beads. CD3^+^ T cells were infected with lentiviral vectors to obtain CD3^+^ T cells with differential Drp1 expression levels: wild-type CD3^+^ T cells (wtT), Drp1-overexpressing T cells (oeDT), Drp1 knockdown T cells (shDT), and corresponding negative control T cells (oeNT and shNT). The proportion of CD3^+^ T cells was confirmed by flow cytometry. The Drp1 protein level was estimated by western blotting assay (Fig. 1).

### Cell co-culture

Before co-culture, CD3^+^ T cells were incubated with monoclonal antibody (mAb) against PD-1 for 24 h to block surface PD-1 receptors. A549 cells were labeled with carboxyfluorescein succinimidyl ester (CFSE). CD3^+^ T cells with different Drp1 expression levels were co-cultured with A549 cells, and PD-1 mAb was added to the groups. Fourteen experimental groups were established (Table S1).

### Assay of T cell cytokines

ELISA kits (MultiSciences Biotech Co., Ltd., Hangzhou, China) were used to measure the concentrations of interferon gamma (IFN-γ), tumor necrosis factor-alpha (TNF-α), perforin, and granzyme B in each group after infection with the lentiviral vectors and contact co-culture.

### Analysis of cell proliferation and death

The proportions of viable and dead A549 cells were detected by flow cytometry. The mean fluorescence intensity (MFI) of CFSE and the number of CFSE^+^PI^+^ cells in each group were detected.

### Lactate dehydrogenase assay to assess cytotoxicity

Lactate dehydrogenase (LDH) levels in all experimental groups were measured using an LDH cytotoxicity detection kit (Beyotime Biotech Co., Ltd., Shanghai, China) to determine the cytotoxicity of CD3^+^ T cells.

### Establishment of a xenograft model

Forty-five nude mice were randomly allocated to one of nine groups (Table S1). After 1 week of adaptive feeding, each mouse was subcutaneously injected with a suspension of A549 cells into the right abdomen to establish a xenograft model. Tumor volume was calculated three times per week according to the following formula: tumor volume = 0.5 × Y × y^2^ [11], where Y is the maximum diameter and y is the minimum diameter (y). Except for the animals in the control group, each mouse was intravenously injected with CD3^+^ T cells at the density of 5 × 10^6^ cells/200 µL. In addition, mice in groups G, H, and I were intraperitoneally injected with 250 µg of PD-1 mAb on days 1, 4, and 7, while mice in the other groups were injected with the same amount of normal saline. All mice were sacrificed at 2 weeks after the last injection, and the tumor and spleen tissues were immediately collected and stored for further analysis.

### Immunofluorescence and immunohistochemical analysis

Immunofluorescence assay was used to detect epithelial-mesenchymal transition (EMT)-related proteins and the PD-L1 protein in cell and animal experiments and the levels of proteins involved in the ERK and Drp1 signaling pathways in cell experiments. Briefly, cell slides and fixed tissue slides were incubated with 0.1% Triton X-100 for 15 min followed by incubation with bovine serum albumin for 15 min. Subsequently, the tissue specimens were incubated with primary antibodies diluted to 1:100 in phosphate-buffered saline (PBS) overnight at 4 °C. On the next day, the specimens were washed with PBS and then incubated with secondary antibodies diluted to 1:200 at room temperature for 60 min. The nuclei were labeled with 4′,6-diamidino-2-phenylindole for 5 min, and a fluorescent quencher was added dropwise prior to observation under a fluorescence microscope.

Paraffin-embedded sections were dewaxed, blocked, incubated first with primary antibodies and then with secondary antibodies (Ki-67 and horseradish peroxidase-conjugated goat anti-rabbit immunoglobulin G, respectively), mounted on glass slides, and observed under a microscope.

### Statistical analysis

All statistical analyses were performed using IBM SPSS Statistics for Windows, version 25.0. (IBM Corporation, Armonk, NY, USA). All analyses were repeated three times. The data are expressed as mean ± standard deviation (SD). Multigroup comparisons were conducted with one-way analysis of variance (ANOVA). The significance of differences in tumor volume among the groups was determined by repeated-measures ANOVA. Kaplan–Meier survival curves were generated. A probability (*p*) value of < 0.05 was considered statistically significant.

## Results

### High levels of Drp1 enhance T cell activity, cytotoxicity, and the inhibitory effect on lung cancer cells

**Fig. 2:**
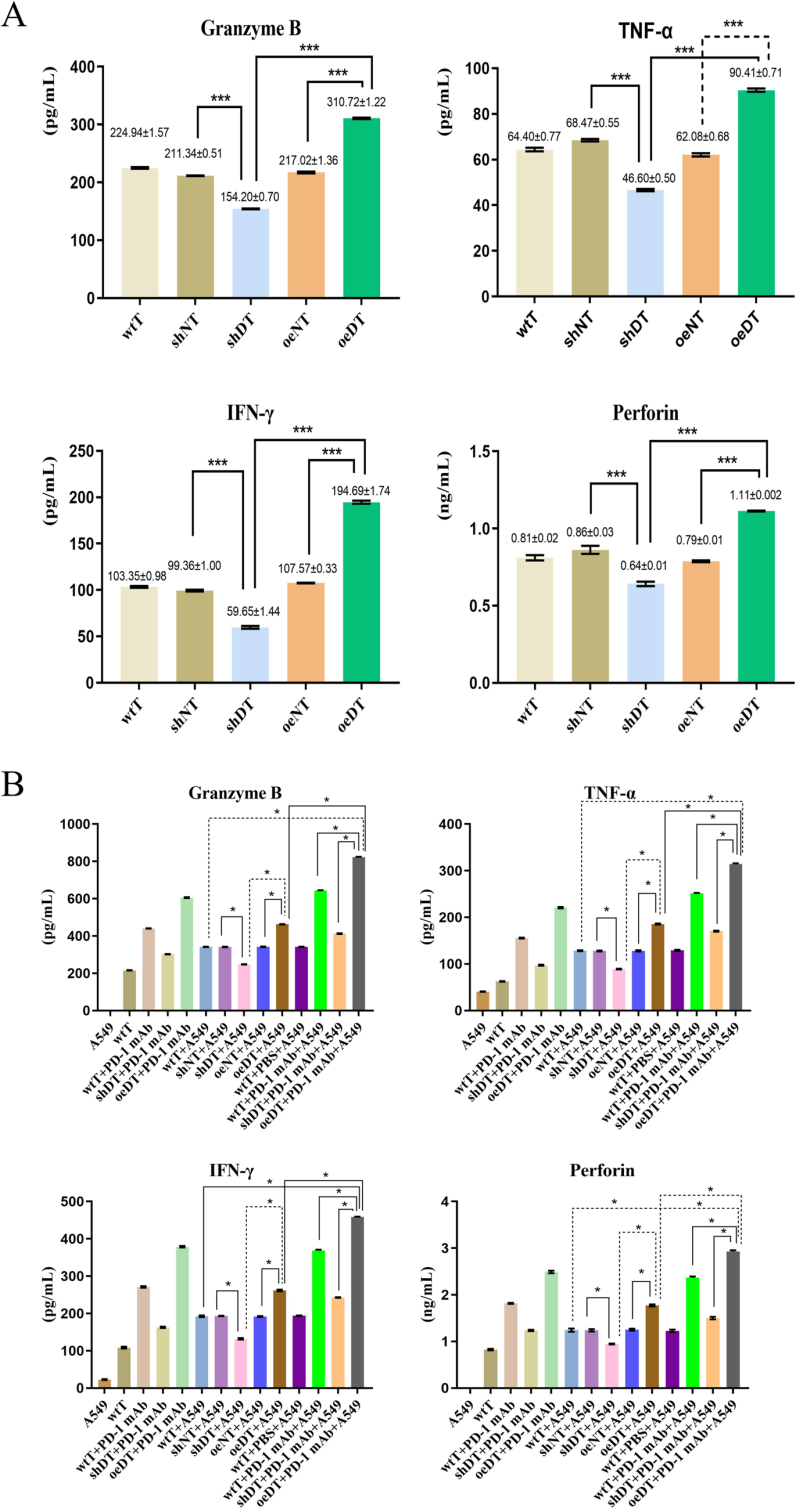
Cytokine secretion levels of T cells with different expression levels of Drp1 in the co-cultured groups Secretion of IFN-γ, granzyme B, perforin, and TNF-α (**A**) by T cells with different Drp1 expression levels and (**B**) in different co-culture groups. ***p < 0.05. Abbreviations: wtT: wild-type CD3^+^ T cells; shDT: CD3^+^ T cells with Drp1 knockdown; oeDT: CD3^+^ T cells with Drp1 overexpression; shNT and oeNT: negative control groups of shDT and oeDT, respectively; PD-1 mAb: programmed cell death protein 1 monoclonal antibody; PBS: phosphate-buffered saline

To investigate whether different Drp1 expression levels affect the function of T cells, we first determined the differences in cytokine secretion among the five groups of T cells with different Drp1 expression levels in vitro. The results showed that compared to the shDT and wtT groups, the oeDT group secreted higher levels of INF-γ, granzyme B, perforin, and TNF-α (as shown in Table S2, Fig. 2A; *p* < 0.05). We co-cultured different groups of T cells with A549 cells (7 groups in total). ELISA and LDH test showed that the levels of T cytokine secretion and LDH in the oeDT co-culture group were significantly higher than those in the shDT and wtT co-culture groups (*p* < 0.05; Table 1 and S3, Fig. 2B).

**Table 1:** Cytokine secretion levels of T cells with different expression levels of Drp1 co-cultured with A549 cells and treated with or without PD-1 mAbs (mean ± SD)

Group	IFN-γ	Granzyme B	Perforin	TNF-α
A549	22.61 ± 1.22	0.00	0.00	40.78 ± 0.51
wtT	108.43 ± 2.56	215.49 ± 1.29	0.82 ± 0.02	62.57 ± 0.70
wtT + PD-1 mAb	271.15 ± 2.17	440.36 ± 0.79	1.82 ± 0.01	155.55 ± 0.90
shDT + PD-1 mAb	162.75 ± 1.86	302.74 ± 1.09	1.24 ± 0.01	97.37 ± 1.09
oeDT + PD-1 mAb	378.96 ± 2.20	605.19 ± 2.42	2.49 ± 0.03	220.73 ± 1.42
wtT + A549^d^	192.68 ± 2.27	341.56 ± 1.15	1.24 ± 0.03	128.57 ± 1.03
shNT + A549^a^	193.43 ± 0.65	340.95 ± 1.61	1.24 ± 0.03	127.90 ± 1.03
shDT + A549^ac^	131.90 ± 2.07	247.45 ± 1.57	0.95 ± 0.01	89.07 ± 0.76
oeNT + A549^b^	191.92 ± 1.13	342.17 ± 1.65	1.25 ± 0.02	128.12 ± 1.40
oeDT + A549^bce^	261.86 ± 2.30	462.54 ± 1.33	1.77 ± 0.02	185.57 ± 1.16
wtT + PBS + A549	193.75 ± 1.17	341.86 ± 1.15	1.23 ± 0.03	129.13 ± 1.36
wtT + PD-1 mAb + A549^f^	368.75 ± 2.18	643.47 ± 1.90	2.36 ± 0.03	251.38 ± 1.07
shDT + PD-1 mAb + A549^g^	242.87 ± 0.86	412.51 ± 1.76	1.50 ± 0.03	170.46 ± 1.13
oeDT + PD-1 mAb + A549^defg^	458.43 ± 1.06	821.69 ± 2.81	2.93 ± 0.03	314.06 ± 1.62

The same superscript letter (e.g., A, B, C) indicates a significant difference between the groups (*p* < 0.001). Abbreviations: wtT: wild-type CD3^+^ T cell; shDT: CD3^+^ T cells with Drp1 knockdown; oeDT: CD3^+^ T cells with Drp1 overexpression; shNT and oeNT: negative control groups of T-shDrp1 and T-oeDrp1, respectively; PD-1 mAb: programmed cell death protein 1 monoclonal antibody; PBS: phosphate-buffered saline

**Fig. 3:**
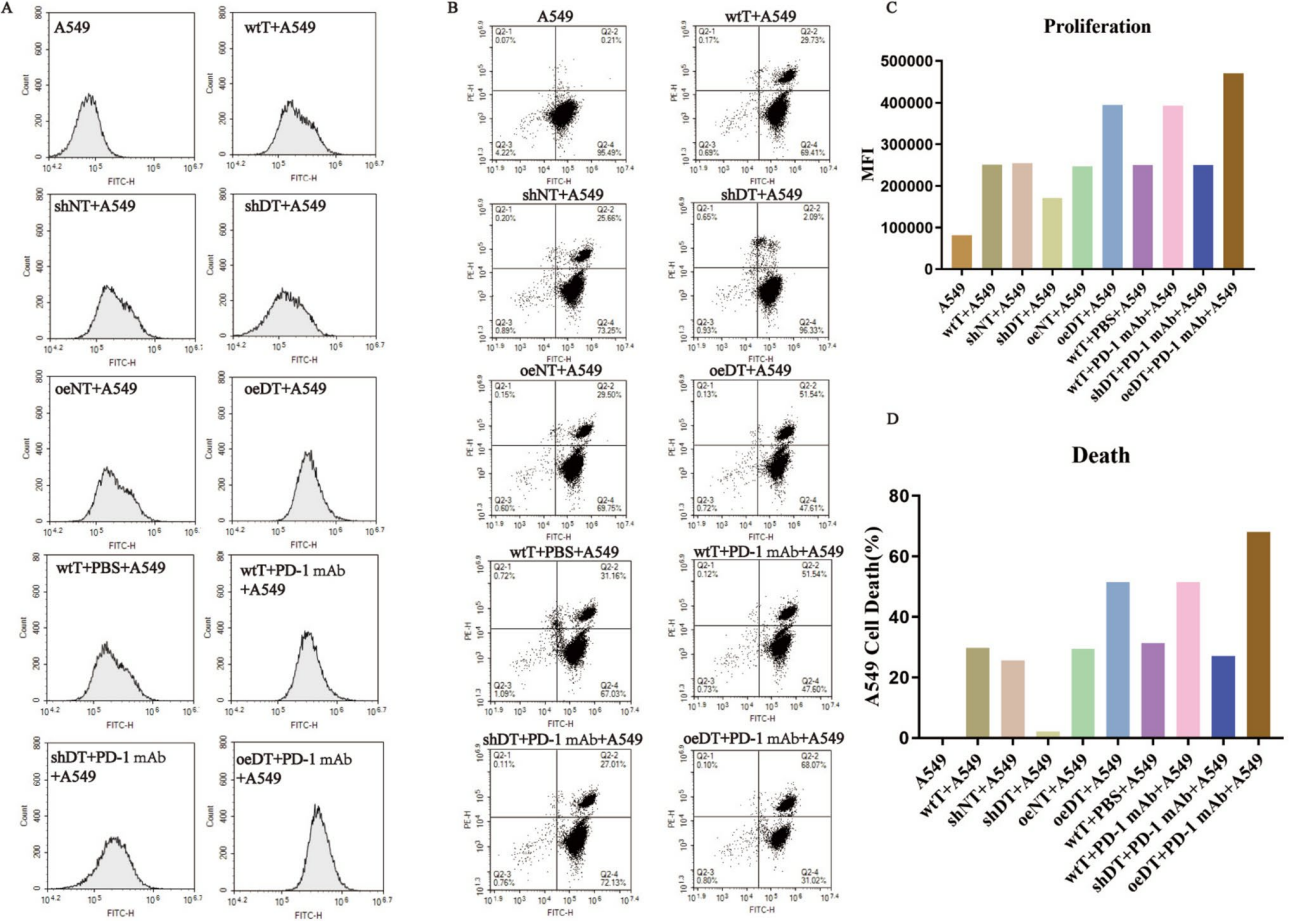
Effects of T cells with different expression levels of Drp1 combined with or without PD-1 mAb on the growth of A549 cells. **A** and **C** Measurement of CFSE fluorescence to assess the proportion of viable A549 cells. **B** and **D** Determination of CFSE-PI dual fluorescence to assess the proportion of dead A549 cells. Abbreviations: wtT: wild-type CD3^+^ T cells; shDT: CD3^+^ T cells with Drp1 knockdown; oeDT: CD3^+^ T cells with Drp1 overexpression; shNT and oeNT: negative control groups of shDT and oeDT, respectively; PD-1 mAb: programmed cell death protein 1 monoclonal antibody; PBS: phosphate-buffered saline

To further understand the effect of Drp1-altered T cells on the proliferation and death of lung cancer cells, we estimated the MFI and death rate of cancer cells in the above-mentioned co-culture groups by flow cytometry assay. The results showed that both the proliferation rate (MFI value) and the death rate of cancer cells were correlated with the Drp1 expression level in T cells. The oeDT co-culture group showed higher growth inhibitory and death rates of A549 cells than the shDT and wtT co-culture groups (Table S4, Fig. 3).

**Fig. 4:**
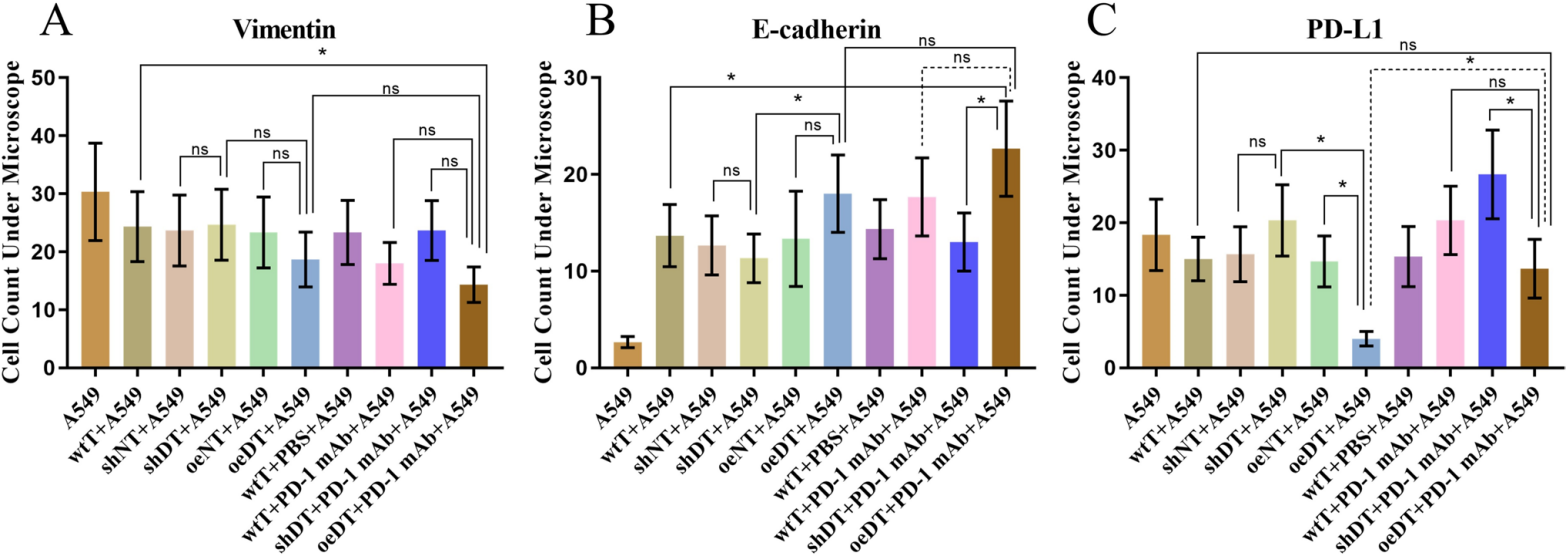
Effects of T cells with different expression levels of Drp1 combined with or without PD-1 mAb on epithelial-mesenchymal transition (EMT) and tumor immune escape of A549 cells. **A** Vimentin and **B** E-cadherin. **C** PD-L1 (immunosuppression-related protein). *p < 0.05; ns, not significant. Abbreviations: wtT: wild-type CD3^+^ T cells; shDT: CD3^+^ T cells with Drp1 knockdown; oeDT: CD3^+^ T cells with Drp1 overexpression; shNT and oeNT: negative control groups of T-shDrp1 and T-oeDrp1, respectively; PD-1 mAb: programmed cell death protein 1 monoclonal antibody; PBS: phosphate-buffered saline

To further clarify whether the EMT and PD-L1 expression were also affected, we detected the levels of E-cadherin, vimentin, and PD-L1 in lung cancer cells from the different co-culture groups by immunofluorescence assay. We observed that E-cadherin was significantly higher in the oeDT co-culture group than in the shDT co-culture group (*p* < 0.05); however, vimentin level was lower in the oeDT co-culture group than in the shDT co-culture group (18.67 ± 4.73 vs. 23.67 ± 6.11), although the difference was not statistically significant. The oeDT co-culture group showed the lowest expression level of PD-L1 in cancer cells; this expression level was significantly lower than that in the shDT co-culture group (*p* < 0.05; Table S5, Figs. 4, S1, S2, and S3). These findings suggest that T cells with high expression levels of Drp1 can inhibit invasive metastasis and immune escape of lung cancer cells.

**Fig. 5:**
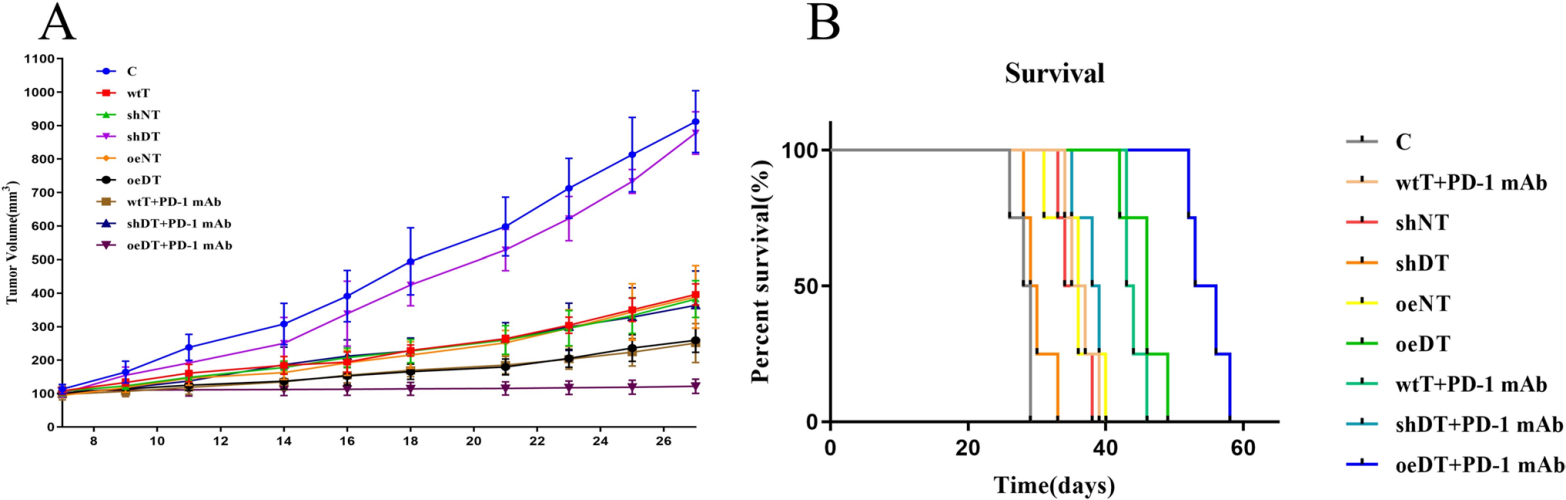
Tumor growth (**A**) and survival curves (**B**) of the nude mouse xenograft models Abbreviations: C: control; wtT: wild-type CD3^+^ T cells; shDT: CD3^+^ T cells with Drp1 knockdown; oeDT: CD3^+^ T cells with Drp1 overexpression; shNT and oeNT: negative control groups of shDT and oeDT, respectively; PD-1 mAb: programmed cell death protein 1 monoclonal antibody

In the nude mouse xenograft model in the adoptive immunotherapy experiments, the tumor volume on day 27 in the oeDT group (259.17 ± 36.48 cm^3^) was smaller than that in the wtT group (396.01 ± 31.763 cm^3^, *p* < 0.001 vs. the shDT group) and the shDT group (877.81 ± 63.40 cm^3^, *p* < 0.001 vs. the oeDT group); moreover, the mean survival time of mice in the oeDT, shDT, and wtT groups was 45.75 ± 2.87, 36.25 ± 2.22, and 30.00 ± 2.16 days, respectively (*p* < 0.001, oeDT group vs. shDT group; shDT group vs. wtT group) (Table S6, Figs. 5 and S4). Immunohistochemical assay showed that Ki-67 expression in the tumor tissues of the oeDT group was significantly lower than that in the shDT and wtT groups (*p* < 0.05; Table S7, Figure S5). Immunofluorescence assay showed that the oeDT adoptive immunotherapy group had the lowest expression level of PD-L1 in the mouse spleen and tumor tissue as well as the lowest enriched number of PD-L1^+^CD8^+^ double-positive T cells; this number was significantly lower than that in the shDT and wtT group (*p* < 0.05; Table 2, Figures S6-8).

**Table 2:** Number of CD8^+^ T cells, PD-L1^+^ cells, and PD-L1^+^CD8^+^ double-positive T cells in the spleen and tumor of each group (mean ± SD)

Group	Spleen			Tumor		
	CD8^+^ T cells	PD-L1^+^ cells	PD-L1^+^CD8^+^ T cells	CD8^+^ T cells	PD-L1^+^ cells	PD-L1^+^ CD8^+^ T cells
C	23.67 ± 6.66	23.00 ± 5.00	18.00 ± 3 0.00	30.00 ± 6.24	31.00 ± 6.56	26.33 ± 5.69
wtT	19.33 ± 6.70^b^	20.00 ± 3.46	18.67 ± 4.16	27.00 ± 6.06^b^	28.67 ± 6.66	24.33 ± 6.03^c^
shNT	20.33 ± 3.37^a^	21.00 ± 6.00	17.00 ± 2.65^a^	20.67 ± 3.37^a^	28.00 ± 4.00	19.67 ± 2.89^a^
shDT	32.00 ± 8.29^a^	29.00 ± 2.65^b^	26.33 ± 3.79^ab^	35.67 ± 9.91^a^	35.67 ± 4.51^b^	33.33 ± 3.79^ab^
oeNT	22.33 ± 4.51	21.67 ± 4.16^a^	20.00 ± 2.65	26.67 ± 7.8	29.00 ± 3.61^a^	23.00 ± 2.65
oeDT	30.33 ± 4.97	14.33 ± 2.52^abc^	14.00 ± 2.65^bc^	32.67 ± 8.85	18.00 ± 3.46^abc^	16.33 ± 4.04^bd^
wtT + PD-1 mAb	35.33 ± 5.51	31.67 ± 6.51	30.00 ± 5.00	38.33 ± 6.99	39.67 ± 5.69	36.33 ± 7.57
shDT + PD-1 mAb	39.33 ± 10.74	34.00 ± 7.21	31.00 ± 5.29	46.00 ± 5.45	55.00 ± 9.00^d^	45.00 ± 6.56^e^
oeDT + PD-1 mAb	36.33 ± 4.20^b^	26.67 ± 4.16^c^	24.00 ± 7.21^c^	41.00 ± 3.56^b^	34.67 ± 4.62^ cd^	33.00 ± 3.61^cde^

The same superscript letter (e.g., a, b, c) indicates a significant difference between the groups (*p* < 0.05). Abbreviations: C: control; wtT: wild-type CD3^+^ T cells; shDT: CD3^+^ T cells with Drp1 knockdown; oeDT: CD3^+^ T cells with Drp1 overexpression; shNT and oeNT: negative control groups of T-shDrp1 and T-oeDrp1, respectively; PD-1 mAb: programmed cell death protein 1 monoclonal antibody; PBS: phosphate-buffered saline

### T cells with a high expression of Drp1 enhance the antitumor immune effects of PD-1 mAbs

Given that T cells expressing high levels of Drp1 showed enhanced inhibitory effects on lung cancer cells and tumors, we further investigated the effect of T cells with different Drp1 expression levels on the antitumor immune response of PD-1 mAbs in the treatment of lung cancer. In vitro cell experiments showed that the T cell activity, cytotoxicity, inhibition of cancer cell proliferation, promotion of cancer cell death, and inhibition of cancer cell invasion and EMT in the oeDT plus pembrolizumab group were significantly higher than those in the oeDT group, wtT plus pembrolizumab group, and shDT plus pembrolizumab group (*p* < 0.05; Figs. 2, 3, 4). The expression level of PD-L1 in cancer cells in the oeDT plus pembrolizumab group was significantly higher than that in the oeDT group, but significantly lower than that in the shDT plus pembrolizumab group (*p* < 0.05); this finding indicated that oeDT combined with PD-1 mAbs can increase the expression of PD-L1 on the surface of cancer cells, promote T cell recognition of cancer cells, and enhance T cell antitumor immune response (Fig. 4).

In the mouse tumor model, the tumor volume on day 27 in the oeDT plus pembrolizumab group (121.94 ± 21.48 cm^3^) was smaller than that in the shDT plus pembrolizumab group (363.84 ± 103 cm^3^, *p* < 0.001). The mean survival time of the oeDT plus pembrolizumab group (54.75 ± 2.75 days) was longer than that of the shDT plus pembrolizumab group (38.00 ± 2.16 days), the wtT group (36.25 ± 2.22 days), and the oeDT group (45.75 ± 2.87 days) (*p* < 0.05; Table S6, Figs. 5 and S4). Immunohistochemical assay revealed that the tumor tissues in the oeDT plus pembrolizumab group showed significantly less Ki-67 expression level than the shDT plus pembrolizumab group (Table S7, Figure S5). The number of infiltrated CD8^+^ T cells in the tumors of the mice in the oeDT plus pembrolizumab group was significantly higher than those in the wtT and control groups (*p* < 0.05). The enrichment of PD-L1^+^CD8^+^ double-positive T cells in tumor tissues was less in the oeDT plus pembrolizumab group than in the shDT plus pembrolizumab group (*p* < 0.05) and the wtT plus pembrolizumab group. The numbers of CD8^+^ T cells and PD-L1^+^CD8^+^ double-positive T cells in the spleen of the oeDT plus pembrolizumab group were also lower than those in the shDT plus pembrolizumab and wtT plus pembrolizumab groups, although the difference was not statistically significant (Table 2, Figures S6–8).

### Modulation of the PD1-ERK/Drp1 pathway synergistically potentiates T-cell antitumor immune response

**Fig. 6:**
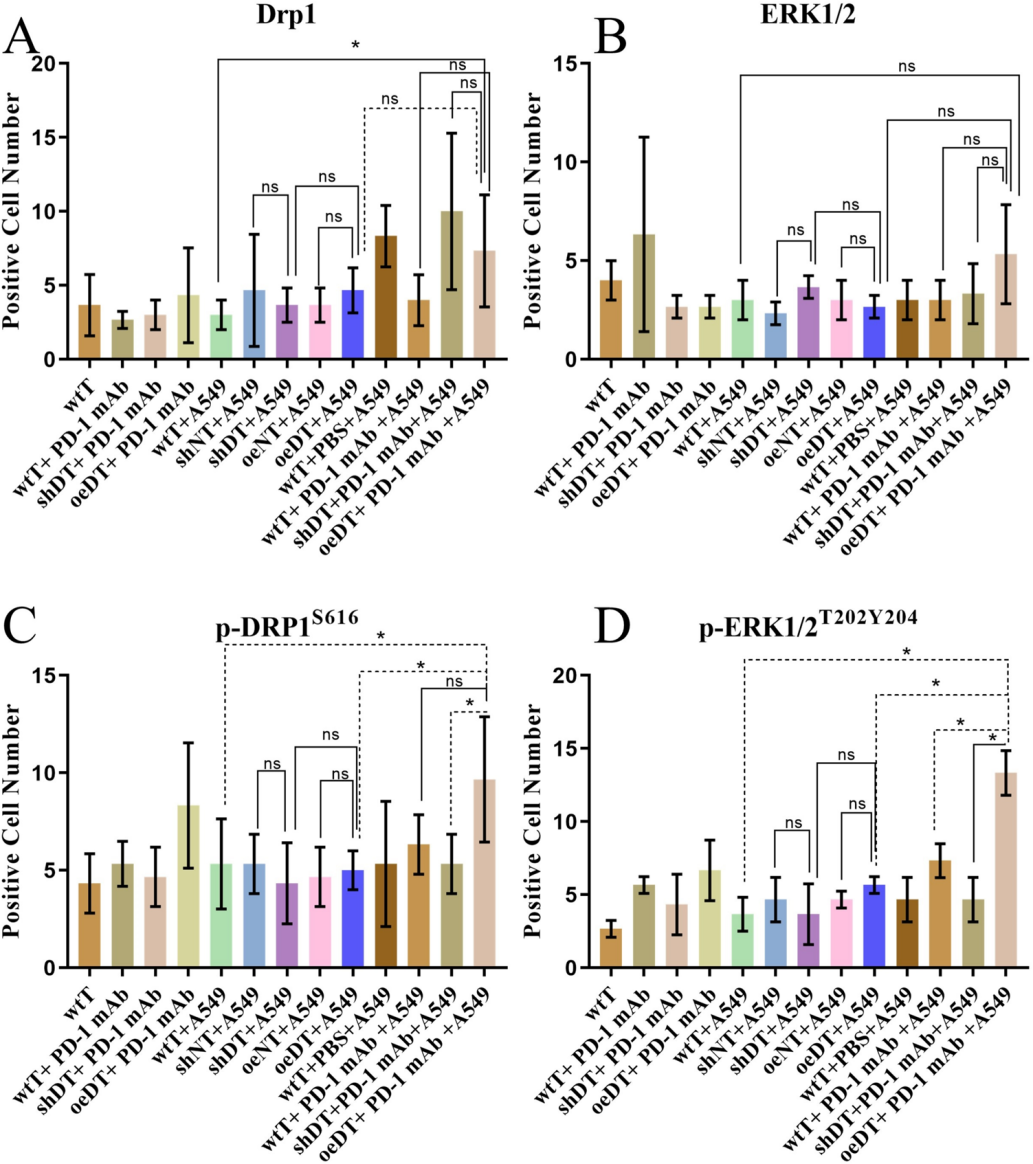
Expression levels of proteins and phosphorylation levels of Drp1 and ERK in co-cultured groups. **A** Drp1. **B** ERK1/2. **C** p-Drp1^s616^. **D** p-ERK1/2^T202Y204^. *p < 0.05; ns, not significant. Abbreviations: wtT: wild-type CD3^+^ T cells; shDT: CD3^+^ T cells with Drp1 knockdown; oeDT: CD3^+^ T cells with Drp1 overexpression; shNT and oeNT: negative control groups of shDT and oeDT, respectively; PD-1 mAb: programmed cell death protein 1 monoclonal antibody; PBS: phosphate-buffered saline

To clarify whether there is crosstalk between the PD-1 signaling pathway and the ERK and Drp1 signaling pathways, we detected Drp1 and ERK pathway proteins such as Drp1, p-Drp1^S616^, ERK1/2, and p-ERK1/2^T202Y204^ by conducting immunofluorescence assay in different co-culture systems of T cells. The results showed no difference in the protein expression levels of Drp1 and ERK1/2 in T cells between the groups. The expression levels of p-Drp1^S616^ and p-ERK1/2^T202Y204^ were significantly higher in the oeDT plus pembrolizumab group than in the shDT plus pembrolizumab group, the oeDT group, and the shDT group (*p* < 0.05); however, no significant difference was observed between the oeDT and shDT groups. Moreover, the expression of p-ERK1/2^T202Y204^ was significantly lower in the shDT plus pembrolizumab group than in the wtT plus pembrolizumab group (*p* < 0.05; Table 3, Figs. 6 and S9-12). This finding suggests that PD-1 mAbs synergize the antitumor immune effect of T cells by promoting ERK or Drp1 expression.

**Table 3:** Expression levels of proteins and phosphorylation level of Drp1 and ERK in the co-cultured groups (mean ± SD)

Group	Drp1	ERK1/2	p-Drp1^S616^	p-ERK1/2^T202Y204^
wtT	3.67 ± 2.08	4.00 ± 1.00	4.33 ± 1.53	2.67 ± 0.58
wtT + PD-1 mAb	2.67 ± 0.58	6.33 ± 4.93	5.33 ± 1.15	5.67 ± 0.58
shDT + PD-1 mAb	3.00 ± 1.00	2.67 ± 0.58	4.67 ± 1.53	4.33 ± 2.08
oeDT + PD-1 mAb	4.33 ± 3.21	2.67 ± 0.58	8.33 ± 3.21	6.67 ± 2.08
wtT + A549	3.00 ± 1.00^a^	3.00 ± 1.00	5.33 ± 2.31^a^	3.67 ± 1.15^a^
shNT + A549	4.67 ± 3.79	2.33 ± 0.58	5.33 ± 1.53	4.67 ± 1.53
shDT + A549	3.67 ± 1.15	3.67 ± 0.58	4.33 ± 2.08	3.67 ± 2.08
oeNT + A549	3.67 ± 1.15	3.00 ± 1.00	4.67 ± 1.53	4.67 ± 0.58
oeDT + A549	4.67 ± 1.53	2.67 ± 0.58	5.00 ± 1.00^b^	5.67 ± 0.58^b^
wtT + PBS + A549	8.33 ± 2.08	3.00 ± 1.00	5.33 ± 3.21	4.67 ± 1.53
wtT + PD-1 mAb + A549	4.00 ± 1.73	3.00 ± 1.00	6.33 ± 1.53	7.33 ± 1.15^c^
shDT + PD-1 mAb + A549	10.00 ± 5.29	3.33 ± 1.53	5.33 ± 1.53^c^	4.67 ± 1.53^d^
oeDT + PD-1 mAb + A549	7.33 ± 3.79^a^	5.33 ± 2.52	9.67 ± 3.21^abc^	13.33 ± 1.53^abcd^

The same superscript letter (e.g., a, b, c) indicates a significant difference between the groups (*p* < 0.05). Abbreviations: wtT: wild-type CD3^+^ T cells; shDT: CD3^+^ T cells with Drp1 knockdown; oeDT: CD3^+^ T cells with Drp1 overexpression; shNT and oeNT: negative control groups of T-shDrp1 and T-oeDrp1, respectively; PD-1 mAb: programmed cell death protein 1 monoclonal antibody; PBS: phosphate-buffered saline

## Discussion

Drp1 plays an important role in regulating the intracellular mitochondrial morphology and function, and it is crucial for the activation and differentiation of T cells into effector T cells [12]. However, the inhibitory effect of Drp1 regulation of T cells on the tumor growth and metastasis of lung cancer as well as the synergistic effect of the antitumor immune effect of Drp1-expressing T cells in combination with anti-PD-1 mAbs are not fully understood. The present study showed that high expression levels of Drp1 significantly enhanced T cell activity and cytotoxicity and synergistically enhanced the anticancer effects of PD-1 mAbs both in vitro and in vivo; this phenomenon was attributed to the promotion of T cell infiltration into the TME and immune organs. Mechanistically, this synergistic effect may be related to the secretion of immune killing-related cytokines and the regulation of the ERK/Drp1 pathways by PD-1 signaling.

Notably, we observed that high expression levels of Drp1 can enhance the activity and toxicity of T cells and inhibit EMT and immune escape of cancer cells. Cytokines (IFN-γ, granzyme B, perforin, and TNF-α) secreted by T cells are important for maintaining immunosurveillance, and inhibition of these cytokines can promote the growth and metastasis of tumor cells [13–18]. The secretion of large amounts of cytokines also promotes further activation of natural killer cells and augment antigen presentation, thereby enhancing the immune response [19]. In the present study, we noted a significant increase in cytokine secretion by oeDT cells and increased death rate of A549 cells in the oeDT and A549 co-culture system, together with the suppression of the EMT phenotype (E-cadherin upregulation and vimentin downregulation); this finding is consistent with the results of the mice adoptive immunotherapy experiments. The inhibition of E-cadherin expression in EMT is characterized by loss of cell adhesion and upregulated expression of the transcription factors snail family transcriptional repressor 1 and 2, forkhead box C1, and vimentin [20]. Thus, T cells regulated by Drp1 may further relieve immunosuppression.

oeDT cells synergistically enhanced the inhibitory effect of PD-1 mAbs in cancer cells. In the in vitro experiment, oeDT combined with PD-1 mAbs exhibited the strongest antitumor effect by inhibiting the growth and proliferation of lung cancer cells. Interestingly, the combination of PD-1 mAbs and shDT cells showed a lower inhibitory effect (killing effect and proliferation inhibition) on A549 cells than wtT cells. These results indicate a synergistic effect between oeDT cells and PD-1 mAbs. PD-1 is an immunosuppressive molecule expressed on the surface of T cells. The interactions between PD-1 and PD-L1 of cancer cells inhibit T cell activation by blocking the signaling of T cell receptors through the activation of the mTOR and MAPK pathways, thereby inhibiting Drp1 activation [21]. PD-1 also directly inhibits glycolysis in exhausted T cells and promotes fatty acid β-oxidation [22]. The PD-1 signaling pathway downregulates the expression of PGC-1α (the most important gene in mitochondrial biogenesis) and promotes mitochondrial polarization, resulting in the accumulation of nonfunctional mitochondria and an imbalance in reactive oxygen species [9, 10]. High levels of Drp1 can reverse the inhibition of T cell metabolism and differentiation and synergize with PD-1 mAbs to enhance the antitumor immune response of T cells. This finding is consistent with the results of Al-Habsi et al., wherein the authors achieved excellent antitumor effects in tumor-bearing mice by combining spermidine and PD-1 inhibitors that enhance mitochondrial metabolism [23]. Spermidine can promote mitochondrial metabolism and enhance mitochondrial respiratory function of T cells by binding to mitochondrial trifunctional proteins.

The mechanism through which oeDT cells and PD-1 mAbs synergistically enhance antitumor activity might be associated with the activation of the PD-1-Erk/Drp1 pathway. Previous studies have shown that the Ras pathway activates Raf-Mek, phosphorylates ERK1/2, and mediates Drp1 phosphorylation at Ser616 to promote mitochondrial fission [24]. The fragmented mitochondrial network relies on the migration of actin and its subsequent redirection to the uropodium or immunological synapse for functioning within the cells [4]. The results of the present study showed that changes in Drp1 expression levels did not significantly alter the expression of proteins involved in the Drp1-related pathways (i.e., ERK/p-ERK and Drp1/p-Drp1); however, significant alterations occurred in the expression of these proteins after PD-1 mAb addition. The expression levels of Drp1, p-Drp1^S616^, and pERK1/2 ^T202Y204^ were significantly higher in the oeDT + PD-1mAb + A549 group than in the wtT + A549 group, with no difference in ERK expression. The synergistic effect of oeDT cells and PD-1 mAbs might be due to the inhibition of the PD-1 signaling pathway by PD-1 mAb blocking through ERK/Drp1. Although alterations in Drp1 expression levels might cause negligible changes to this pathway under the influence of A549 cells, the addition of PD-1 mAbs reversed the inhibitory effect. Alternatively, the lack of changes in Drp1 levels alone might function as a crosstalk among multiple signaling pathways. Previous studies have shown that Drp1 knockdown inhibits T cell migration, but does not affect positive selection of T cells in the thymus; this is because several signaling pathways are involved in cellular apoptosis in the thymic cortex [25]. Ser616 phosphorylation and Ser637 dephosphorylation also promote Drp1 activation, thus indicating that mitochondrial dynamics are regulated by multiple signaling pathways that are crucial for maintaining organelle function [26]. These findings indicate that PD-1 and PD-L1 participate in the regulation of ERK phosphorylation. By blocking this site, PD-1 mAbs increase the p-ERK level and further activate the downstream molecule Drp1.

The antitumor activities of oeDT cells were significantly enhanced in vivo and were further improved by PD-1 mAbs. Interestingly, although the tumor volume on day 27 was 34% smaller in the oeDT group than in the wtT group, no significant difference was noted in the growth curves of the two groups. Combined with previous studies on the polarization and nonfunctional mitochondria of exhausted T cells in the TME, the mitochondrial function of T cells in the TME must be functionally defective, and shDT cells are closer to the functional state of T cells in the TME. The antitumor activities of shDT cells were also reduced in vivo, while the combination of shDT cells with PD-1 mAbs led to nonsignificant improvement. The results were consistent in terms of tumor size, survival time, and expression of the tumor proliferation marker Ki-67. T cells cannot easily penetrate solid tumors because of the highly heterogeneous blood vessels and dense extracellular matrix [27–29]. Hypoxia [30] and continuous stimulation of T cell receptors [5] in the TME will gradually alter the phenotype of T cells to exhausted T cells, which have unique metabolic characteristics [31]. Most tumor-infiltrating lymphocytes show the accumulation of depolarized and nonfunctional mitochondria [5]. In a study of Simula et al. [21], the efficacy of PD-1 mAbs was 60% in the control group, but only 20% in the Drp1 deletion group. Another possible reason is that the promotion of mitochondrial division through Drp1 overexpression may promote the conversion of the metabolic mode to glycolysis and thus affect the survival time of T cells. Although T cell activation is enhanced, accelerated cellular apoptosis prevents T cells from participating in the immune response in a sustained manner [12].

In our present study, oeDT cells combined with PD-1 mAbs improved the proliferation, migration, and infiltration ability of T cells; increased the number of CD8^+^ T cells; and decreased enrichment of PD-L1^+^CD8^+^ double-positive T cells in the spleen and tumor tissues of nude mice. Although the tumor and spleen tissues of the shDT plus PD-1 mAb group had the highest proportion of CD8^+^ T cells, the shDT plus PD-1 mAb group had a significantly higher number of PD-L1^+^CD8^+^ T cells than the oeDT plus PD-1 mAb group. This finding confirmed that most of the CD8^+^ cells in the shDT plus PD-1 mAb group had a high expression of PD-L1. A previous study reported that high infiltration of PD-L1^+^CD8^+^ T cells in the microenvironment of NSCLC represents an immunosuppressive “hot” tumor, accompanied by a higher tumor mutation load, which is related to a better immunotherapeutic effect [32]. However, other studies reported that the PD-L1^−^CD8^+^ phenotype was associated with a better prognosis [33]. These results showed that oeDT plus PD-1 mAbs not only enabled T cells to infiltrate tumor tissues but also prevented immune domestication in the tumor immune microenvironment, thereby avoiding transformation into exhausted T cells. As the largest secondary lymphoid organ in the body, the spleen contains various lymphocytes and immune factors [34]. In the present study, the oeDT plus PD-1 mAb approach increased CD8^+^ T cell infiltration in the spleen, which is critical for splenic immune cells to enter the blood for immune surveillance. High levels of Drp1 could also promote the migration and proliferation of CD8^+^ T cells. Moreover, an increase in the number of CD8^+^ T cells can increase the secretion of cytokines in the spleen. A previous study showed that radiotherapy increased the expression of cytokine IL-1β in the spleen and slowed down tumor growth in a mouse model [35]. Therefore, an increase in splenic T cells may increase cytokine secretion and improve the antitumor effects of the therapy.

Presently, several studies have shown progression in understanding metabolic processes by targeting mitochondria. Myeloid-derived suppressor cells and regulatory cells are targeted with mitochondrial OXPHOS (oxidative phosphorylation) inhibitors to enhance immune efficacy [36, 37]. The ability of CD8^+^ T cells to inhibit tumor cells can be enhanced by overexpression of BH4 (tetrahydrobiopterin), which is required for CD8^+^ T cell expansion [38]. There are several promising targets for enhanced tumor inhibition by T cells, and more research is needed to translate these targets into clinical applications based on efficacy and safety. The transfer of energy metabolism balance to immunogenic cells and the avoidance of energy imbalance caused by regulatory metabolism are the major obstacles to be overcome in future studies.

## Conclusion

High levels of Drp1 enhanced the activities and immune response of CD8^+^ T cells by promoting migration and tumor infiltration and by enhancing the efficacy of PD-1 mAbs. This synergistic mechanism of Drp1- and PD-1-mediated antitumor immune activities of CD8^+^ T cells might be related to the activation of the PD-1-ERK/Drp1 pathway. Hence, further studies are required to assess the antitumor treatment strategies based on targeting the metabolism, migration, and other cellular processes of immune cells, combined with existing treatments (e.g., immunotherapy, radiotherapy, and chemotherapy).

### Supplementary Information

Below is the link to the electronic supplementary material.

Supplementary file 1 (DOCX 30 kb)

Supplementary file 2 (PDF 6993 kb)

## Data Availability

All data and material are available within the article.
