# Supplementary material for: Enhanced T cell immune activity mediated by Drp1 promotes the efficacy of PD-1 inhibitors in treating lung cancer
Source: Cancer Immunol Immunother. 2024 Feb 10;73(2):40. doi: 10.1007/s00262-023-03582-5 (PMC10858821; doi:10.1007/s00262-023-03582-5)
Supplement: Supplementary file 1 — (DOCX 30 kb) [file 262_2023_3582_MOESM1_ESM.docx]

Table S1. Groups names for *in vitro* cell experiments and *in vivo* xenograft experiments.

| *In vitro* experiments | *In vivo* experiments |
| --- | --- |
| A549 | C |
| wtT | wtT |
| wtT+PD-1 mAb | shNT |
| shDT+PD-1 mAb | shDT |
| oeDT+PD-1 mAb | oeNT |
| wtT+A549 | oeDT |
| shNT+A549 | wtT+P |
| shDT+A549 | shDT+P |
| oeNT+A549 | oeDT+P |
| oeDT+A549 |  |
| wtT+PBS+A549 |  |
| wtT+PD-1 mAb +A549 |  |
| shDT+PD-1 mAb +A549 |  |
| oeDT+PD-1 mAb+A549 |  |

Abbreviations: C: control; wtT: wild-type CD3^+^ T cells; shDT: CD3^+^ T cells with Drp1 knockdown; oeDT: CD3^+^ T cells with Drp1 overexpression; shNT and oeNT: negative control groups of shDT and oeDT, respectively; PD-1 mAb: programmed cell death protein 1 monoclonal antibody; PBS: phosphate-buffered saline.

Table S2. Cytokine secretion levels of T cells with different expression levels of Drp1 (mean ± SD).

| Group | IFN-γ | Granzyme B | Perforin | TNF-α |
| --- | --- | --- | --- | --- |
| wtT | 103.35 ± 0.98 | 224.94 ± 1.57 | 0.81 ± 0.02 | 64.40 ± 0.77 |
| shNT^a^ | 99.36 ± 1.00 | 211.34 ± 0.51 | 0.86 ± 0.03 | 68.47 ± 0.55 |
| shDT^ab^ | 59.65 ± 1.44 | 154.20 ± 0.70 | 0.64 ± 0.01 | 46.60 ± 0.50 |
| oeNT^c^ | 107.57 ± 0.33 | 217.02 ± 1.36 | 0.79 ± 0.01 | 62.08 ± 0.68 |
| oeDT^bc^ | 194.69 ± 1.74 | 310.72 ± 1.22 | 1.11 ± 0.002 | 90.41 ± 0.71 |

The same superscript letter (e.g., A, B, C) indicates a significant difference between the groups (*p* < 0.001). Abbreviations: wtT: wild-type CD3^+^ T cells; shDT: CD3^+^ T cells with Drp1 knockdown; oeDT: CD3^+^ T cells with Drp1 overexpression; shNT and oeNT: negative control groups of shDT and oeDT, respectively.

Table S3. Cytotoxicity of T cells among the co-cultured groups (mean ± SD).

| Group | cytotoxicity (%) |
| --- | --- |
| wtT+A549^d^ | 10.10 ± 0.53 |
| shNT+A549^a^ | 10.56 ± 0.91 |
| shDT+A549^ab^ | 7.00 ± 0.72 |
| oeNT+A549^c^ | 10.22 ± 1.38 |
| oeDT+A549^bce^ | 12.51 ± 2.10 |
| wtT+PBS+A549 | 10.45 ± 0.53 |
| wtT+PD-1 mAb+A549^f^ | 17.57 ± 0.87 |
| shDT+PD-1 mAb+A549^g^ | 12.28 ± 2.07 |
| oeDT+PD-1 mAb+A549^defg^ | 20.09 ± 1.55 |

The same superscript letter (e.g., a, b, c) indicates a significant difference between the groups (*p* < 0.05). Abbreviations: wtT: wild-type CD3^+^ T cells; shDT: CD3^+^ T cells with Drp1 knockdown; oeDT: CD3^+^ T cells with Drp1 overexpression; shNT and oeNT: negative control groups of shDT and oeDT, respectively; PD-1 mAb: programmed cell death protein 1 monoclonal antibody; PBS: phosphate-buffered saline.

Table S4. Proliferation and death rates of A549 cells in the co-cultured groups.

| Group | MFI | Death (%) |
| --- | --- | --- |
| A549 | 81435 | 0.21 |
| wtT+A549 | 250874 | 29.73 |
| shNT+A549 | 254054 | 25.66 |
| shDT+A549 | 170609 | 2.09 |
| oeNT+A549 | 247016 | 29.5 |
| oeDT+A549 | 394225 | 51.54 |
| wtT+PBS+A549 | 250195 | 31.36 |
| wtT+PD-1 mAb+A549 | 392704 | 51.54 |
| shDT+PD-1 mAb+A549 | 250240 | 27.01 |
| oeDT+PD-1 mAb+A549 | 470416 | 68.07 |

Abbreviations: wtT: wild-type CD3^+^ T cells; shDT: CD3^+^ T cells with Drp1 knockdown; oeDT: CD3^+^ T cells with Drp1 overexpression; shNT and oeNT: negative control groups of shDT and oeDT, respectively; PD-1 mAb: programmed cell death protein 1 monoclonal antibody; PBS: phosphate-buffered saline.

| Group | E-cadherin | Vimentin | PD-L1 |
| --- | --- | --- | --- |
| A549 | 2.67 ± 0.58 | 30.33 ± 8.39 | 18.33 ± 4.93 |
| wtT+A549 | 13.67 ± 3.21^b^ | 24.33 ± 6.03^a^ | 15.00 ± 3.00 |
| shNT+A549 | 12.67 ± 3.06 | 23.67 ± 6.11 | 15.67 ± 3.79 |
| shDT+A549 | 11.33 ± 2.52^a^ | 24.67 ± 6.11 | 20.33 ± 4.93^b^ |
| oeNT+A549 | 13.33 ± 4.93 | 23.33 ± 6.11 | 14.67 ± 3.51^a^ |
| oeDT+A549 | 18.00 ± 4.00^a^ | 18.67 ± 4.73 | 4.00 ± 1.00^abc^ |
| wtT+PBS+A549 | 14.33 ± 3.06 | 23.33 ± 5.51 | 15.33 ± 4.16 |
| wtT+PD-1 mAb +A549 | 17.67 ± 4.04 | 18.00 ± 3.61 | 20.33 ± 4.73 |
| shDT+PD-1 mAb +A549 | 13.00 ± 3.00^c^ | 23.67 ± 5.13 | 26.67 ± 6.11^d^ |
| oeDT+PD-1 mAb +A549 | 22.67 ± 4.93^bc^ | 14.33 ± 3.06^a^ | 13.67 ± 4.04^cd^ |

Table S5. Expression levels of E-cadherin, Vimentin, and PD-L1 in A549 cells co-cultured with T cells with different expression levels of Drp1 and treated with or without PD-1 mAbs (mean ± SD).

The same superscript letter (e.g., a, b, c) indicates a significant difference between the groups (*p* < 0.05). Abbreviations: wtT: wild-type CD3^+^ T cell; shDT: CD3^+^ T cells with Drp1 knockdown; oeDT: CD3^+^ T cells with Drp1 overexpression; shNT and oeNT: negative control groups of shDT and oeDT, respectively; PD-1 mAb: programmed cell death protein 1 monoclonal antibody; PBS: phosphate-buffered saline.

Table S6. Tumor volume (mm^3^) of the xenograft model in each treatment group (mean ± SD).

| Time  (day) | C | wtT^c^ | shNT^b^ | shDT^ab^ | oeNT | oeDT^a^ | wtT+PD-1 mAb | shDT+PD-1 mAb^d^ | oeDT+PD-1 mAb^cd^ |
| --- | --- | --- | --- | --- | --- | --- | --- | --- | --- |
| 7 | 113.21 ± 14.21 | 108.17 ± 11.73 | 106.86 ± 13.67 | 100.70 ± 11.03 | 94.56 ± 3.35 | 100.88 ± 8.48 | 97.59 ± 16.70 | 100.39 ± 12.96 | 108.29 ± 18.18 |
| 9 | 164.37 ± 32.78 | 132.95 ± 10.62 | 123.06 ± 10.01 | 154.76 ± 24.53 | 118.15 ± 13.9 | 113.54 ± 10.35 | 106.62 ± 15.92 | 116.51 ± 17.63 | 110.05 ± 18.41 |
| 11 | 238.19 ± 38.98 | 161.26 ± 26.32 | 148.03 ± 13.32 | 191.69 ± 42.43 | 145.63 ± 21.13 | 125.01 ± 15.85 | 118.68 ± 20.78 | 137.45 ± 21.33 | 111.49 ± 18.47 |
| 14 | 308.07 ± 62.11 | 184.14 ± 26.79 | 177.92 ± 19.3 | 250.29 ± 77.49 | 162.58 ± 20.45 | 137.08 ± 24.04 | 135.19 ± 24.27 | 186.14 ± 52.65 | 112.18 ± 18.08 |
| 16 | 391.30 ± 76.80 | 194.13 ± 30.79 | 207.42 ± 28.72 | 338.63 ± 97.41 | 191.81 ± 37.47 | 152.80 ± 26.94 | 154.50 ± 22.79 | 212.22 ± 48.61 | 112.84 ± 18.20 |
| 18 | 495.01 ± 100.05 | 228.86 ± 16.57 | 226.87 ± 34.76 | 424.80 ± 62.52 | 215.06 ± 39.95 | 165.61 ± 23.14 | 169.33 ± 18.95 | 227.39 ± 39.19 | 114.11 ± 19.29 |
| 21 | 598.37 ± 88.08 | 263.70 ± 9.96 | 260.17 ± 43.05 | 528.36 ± 61.36 | 251.54 ± 37.19 | 179.69 ± 23.77 | 185.15 ± 26.23 | 264.19 ± 47.86 | 115.41 ± 19.75 |
| 23 | 712.49 ± 89.53 | 304.45 ± 24.35 | 295.63 ± 52.7 | 622.11 ± 66.39 | 295.61 ± 55.11 | 205.25 ± 26.64 | 202.93 ± 29.83 | 299.33 ± 70.84 | 117.17 ± 20.40 |
| 25 | 813.31 ± 111.00 | 350.38 ± 34.89 | 333.20 ± 53.56 | 733.31 ± 35.84 | 344.16 ± 84.56 | 236.04 ± 39.67 | 223.45 ± 41.48 | 328.31 ± 87.91 | 118.91 ± 20.89 |
| 27 | 912.09 ± 92.21 | 396.01 ± 31.76 | 382.68 ± 55.24 | 877.81 ± 63.40 | 388.79 ± 93.76 | 259.17 ± 36.48 | 251.01 ± 58.16 | 363.84 ± 103.00 | 121.94 ± 21.48 |

The same superscript letter (e.g., a, b, c) indicates a significant difference between the groups (*p* < 0.05). Abbreviations: C: control; wtT: wild-type CD3^+^ T cells; shDT: CD3^+^ T cells with Drp1 knockdown; oeDT: CD3^+^ T cells with Drp1 overexpression; shNT and oeNT: negative control groups of shDT and oeDT, respectively; PD-1 mAb: programmed cell death protein 1 monoclonal antibody.

Table S7. Number of Ki-67^+^ cells in each group (mean ± SD).

| Group | Ki-67 |
| --- | --- |
| C | 142.33 ± 24.01 |
| wtT^ab^ | 115.33 ± 21.39 |
| shNT | 113.67 ± 14.57 |
| shDT^c^ | 123.00 ± 16.70 |
| oeNT | 115.00 ± 14.93 |
| oeDT^ac^ | 74.00 ± 24.56 |
| wtT+PD-1 mAb | 68.00 ± 12.49 |
| shDT+PD-1 mAb^d^ | 115.67 ± 16.5 |
| oeDT+PD-1 mAb^bd^ | 33.33 ± 7.64 |

The same superscript letter (e.g., a, b, c) indicates a significant difference between the groups (*p* < 0.05). Abbreviations: C: control; wtT: wild-type CD3^+^ T cells; shDT: CD3^+^ T cells with Drp1 knockdown; oeDT: CD3^+^ T cells with Drp1 overexpression; shNT and oeNT: negative control groups of shDT and oeDT, respectively; PD-1 mAb: programmed cell death protein 1 monoclonal antibody
