# Supplementary material for: Enhanced T cell immune activity mediated by Drp1 promotes the efficacy of PD-1 inhibitors in treating lung cancer
Source: Cancer Immunol Immunother. 2024 Feb 10;73(2):40. doi: 10.1007/s00262-023-03582-5 (PMC10858821; doi:10.1007/s00262-023-03582-5)
Supplement: Supplementary file 2 — (PDF 6993 kb) [file 262_2023_3582_MOESM2_ESM.pdf]

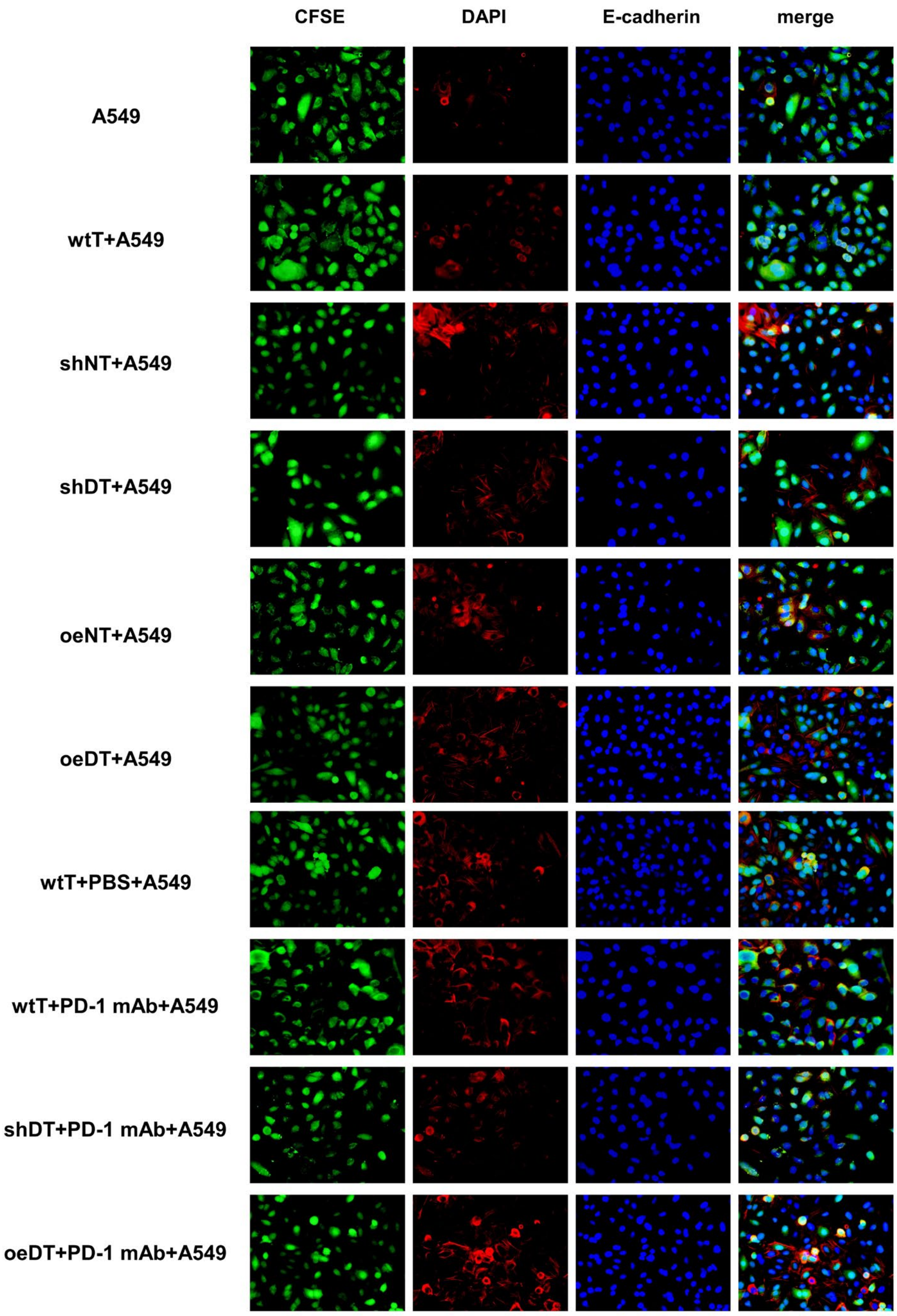

Fig.S1 Immunofluorescence images of E-cadherin expression in A549 cells in the co-culture groups

Abbreviations: wtT: wild-type CD3$^+$ T cells; shDT: CD3$^+$ T cells with Drp1 knockdown; oeDT: CD3$^+$ T cells with Drp1 overexpression; shNT and oeNT: negative control groups of shDT and oeDT, respectively; PD-1 mAb: programmed cell death protein 1 monoclonal antibody; PBS: phosphate-buffered saline.

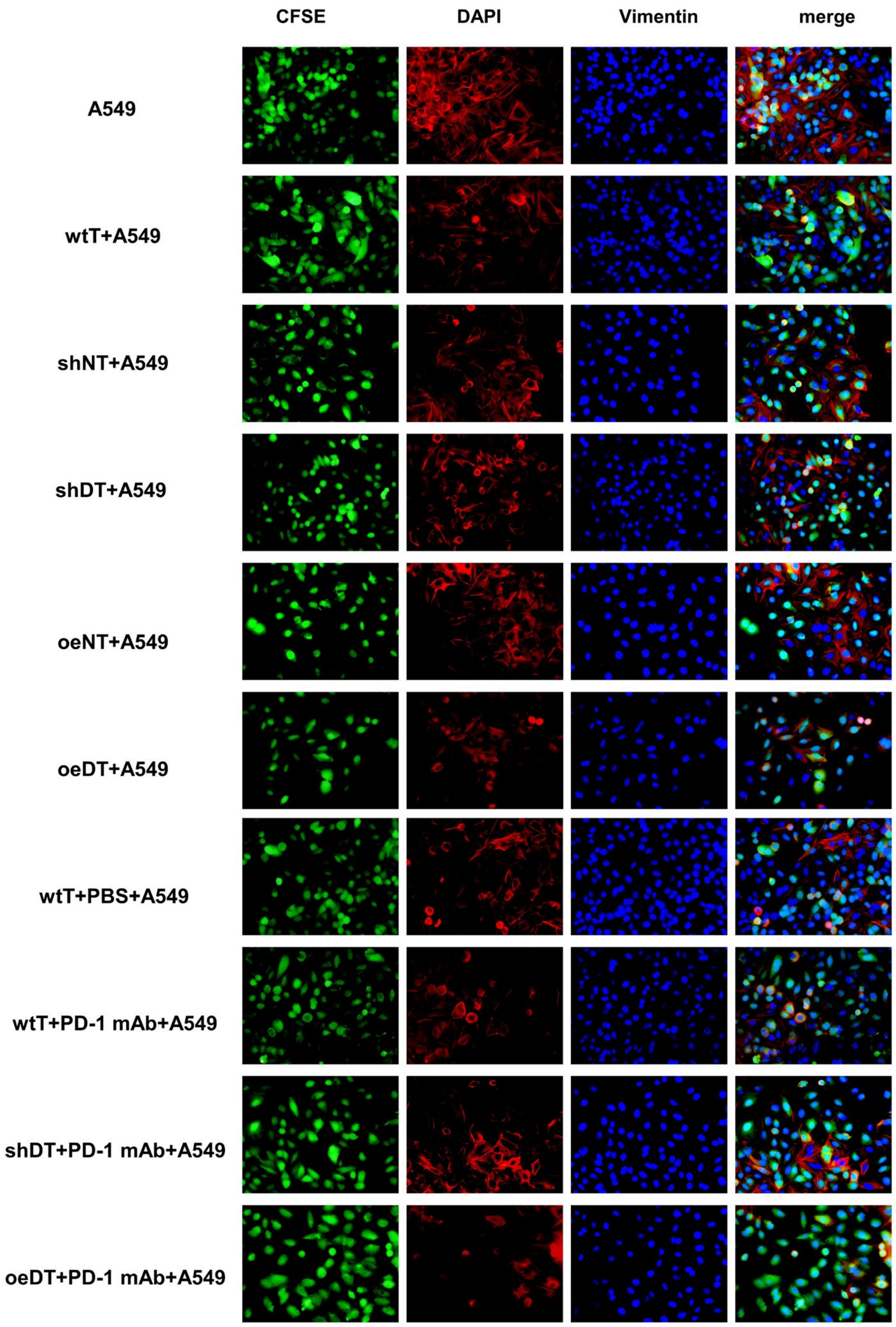

Fig.S2 Immunofluorescence images of vimentin expression in A549 cells in the co-culture groups

Abbreviations: wtT: wild-type $CD3^+$ T cells; shDT: $CD3^+$ T cells with Drp1 knockdown; oeDT: $CD3^+$ T cells with Drp1 overexpression; shNT and oeNT: negative control groups of shDT and oeDT, respectively; PD-1 mAb: programmed cell death protein 1 monoclonal antibody; PBS: phosphate-buffered saline.

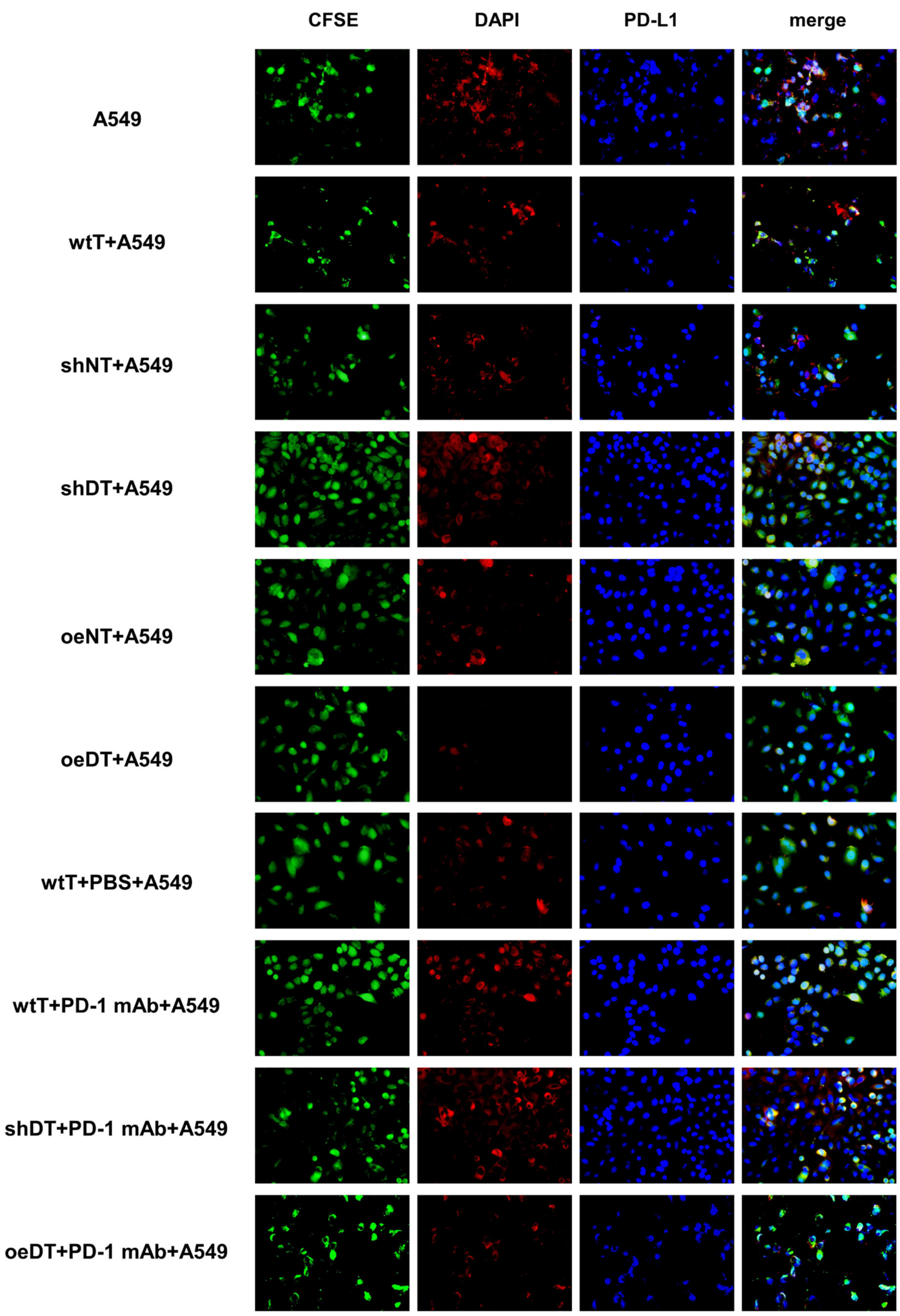

Fig.S3 Immunofluorescence images of PD-L1 expression in A549 cells in the co-culture groups

Abbreviations: wtT: wild-type $CD3^+$ T cells; shDT: $CD3^+$ T cells with Drp1 knockdown; oeDT: $CD3^+$ T cells with Drp1 overexpression; shNT and oeNT: negative control groups of shDT and oeDT, respectively; PD-1 mAb: programmed cell death protein 1 monoclonal antibody; PBS: phosphate-buffered saline.

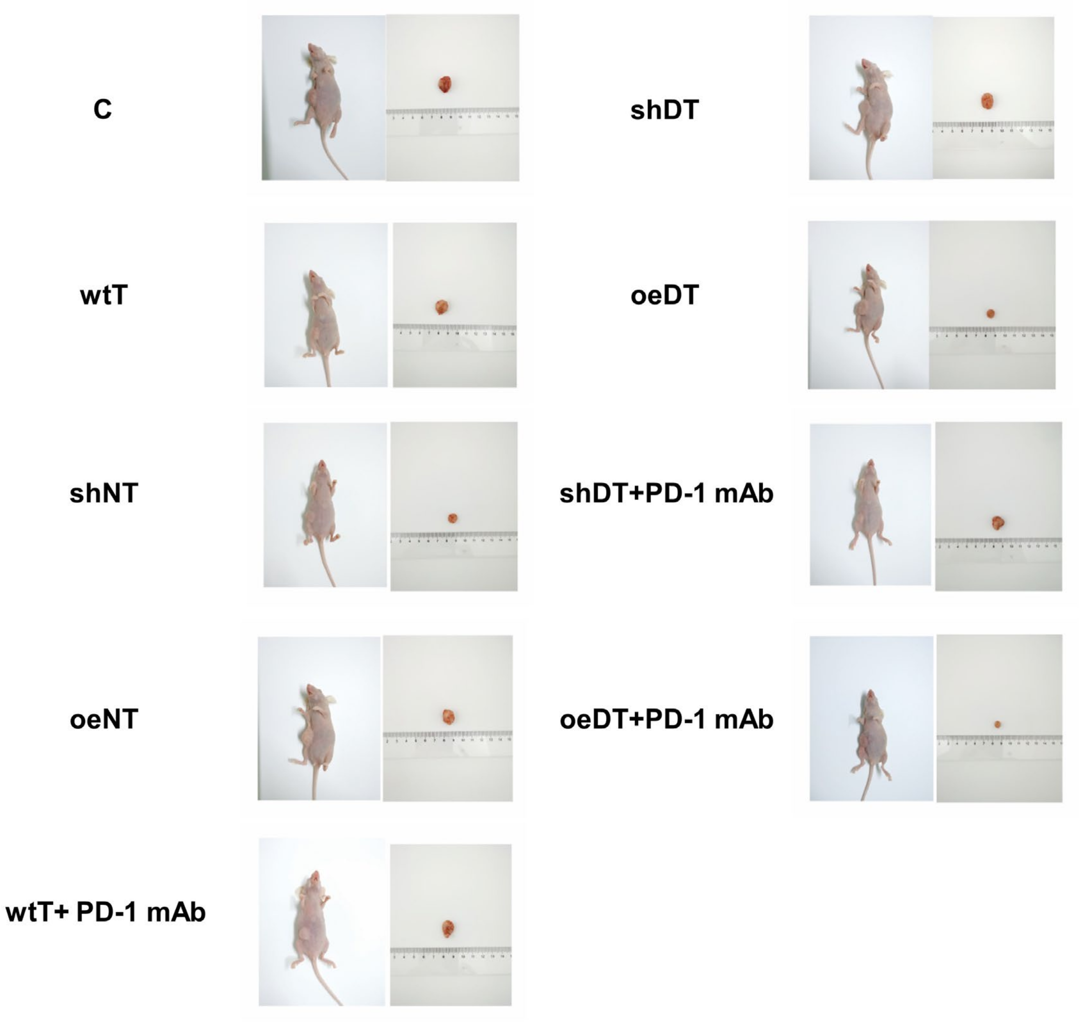

Fig.S4 Growth of tumors in nude mice of each group treated with the combination of T cells with different Drp1 expression levels and PD-1 mAb

Abbreviations: C: control; wtT: wild-type $CD3^+$ T cells; shDT: $CD3^+$ T cells with Drp1 knockdown; oeDT: $CD3^+$ T cells with Drp1 overexpression; shNT and oeNT: negative control groups of shDT and oeDT, respectively; PD-1 mAb: programmed cell death protein 1 monoclonal antibody.

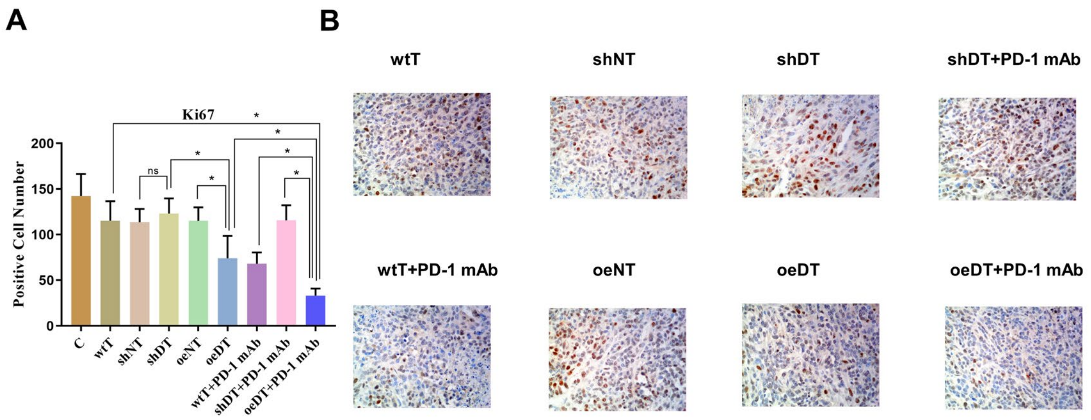

Fig.S5 (A) Ki-67^{+} expression level in each group. (B) Immunohistochemical images $^{*}p < 0.05$; ns, not significant. Abbreviations: C: control; wtT: wild-type CD3^{+} T cells; shDT: CD3^{+} T cells with Drp1 knockdown; oeDT: CD3^{+} T cells with Drp1 overexpression; shNT and oeNT: negative control groups of shDT and oeDT, respectively; PD-1 mAb: programmed cell death protein 1 monoclonal antibody.

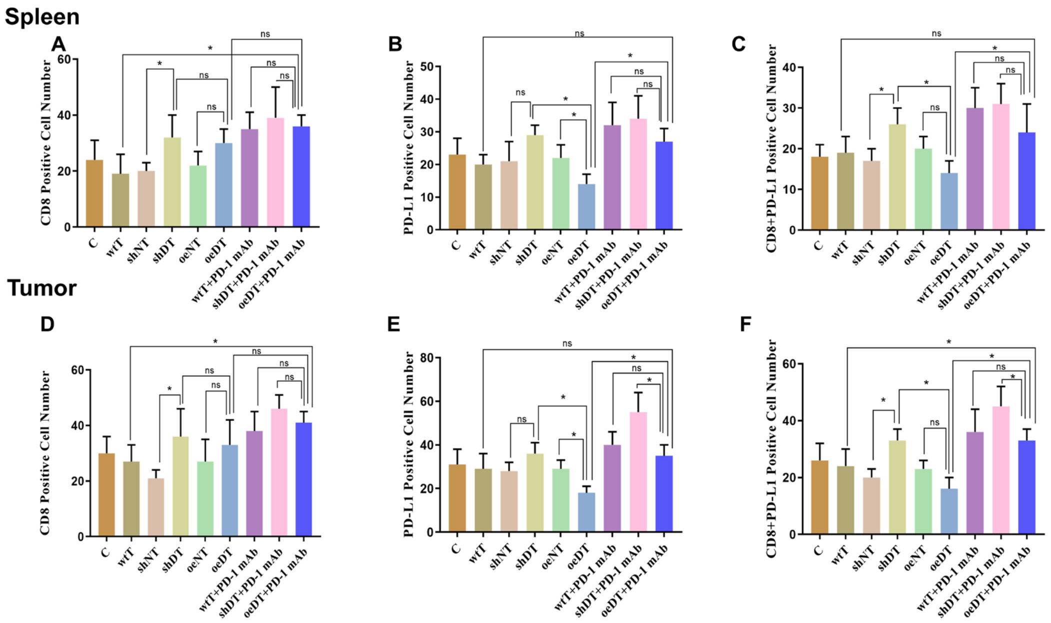

Fig.S6 Proportions of CD8^{+} T and PD-L1^{+} cells in mouse spleen and tumor tissues

(A) Number of CD8^{+} T cells in spleen tissues. (B) Number of PD-L1^{+} cells in spleen tissues. (C) Number of PD-L1^{+}CD8^{+} double-positive T cells in spleen tissues. (D) Number of CD8^{+} T cells in tumor tissues. (E) Number of PD-L1^{+} cells in tumor tissues. (F) Number of PD-L1^{+}CD8^{+} double-positive T cells in tumor tissues. $^{*}p < 0.05$; ns, not significant. Abbreviations: C: control; wtT: wild-type CD3^{+} T cells; shDT: CD3^{+} T cells with Drp1 knockdown; oeDT: CD3^{+} T cells with Drp1 overexpression; shNT and oeNT: negative control groups of shDT and oeDT, respectively; PD-1 mAb: programmed cell death protein 1 monoclonal antibody.

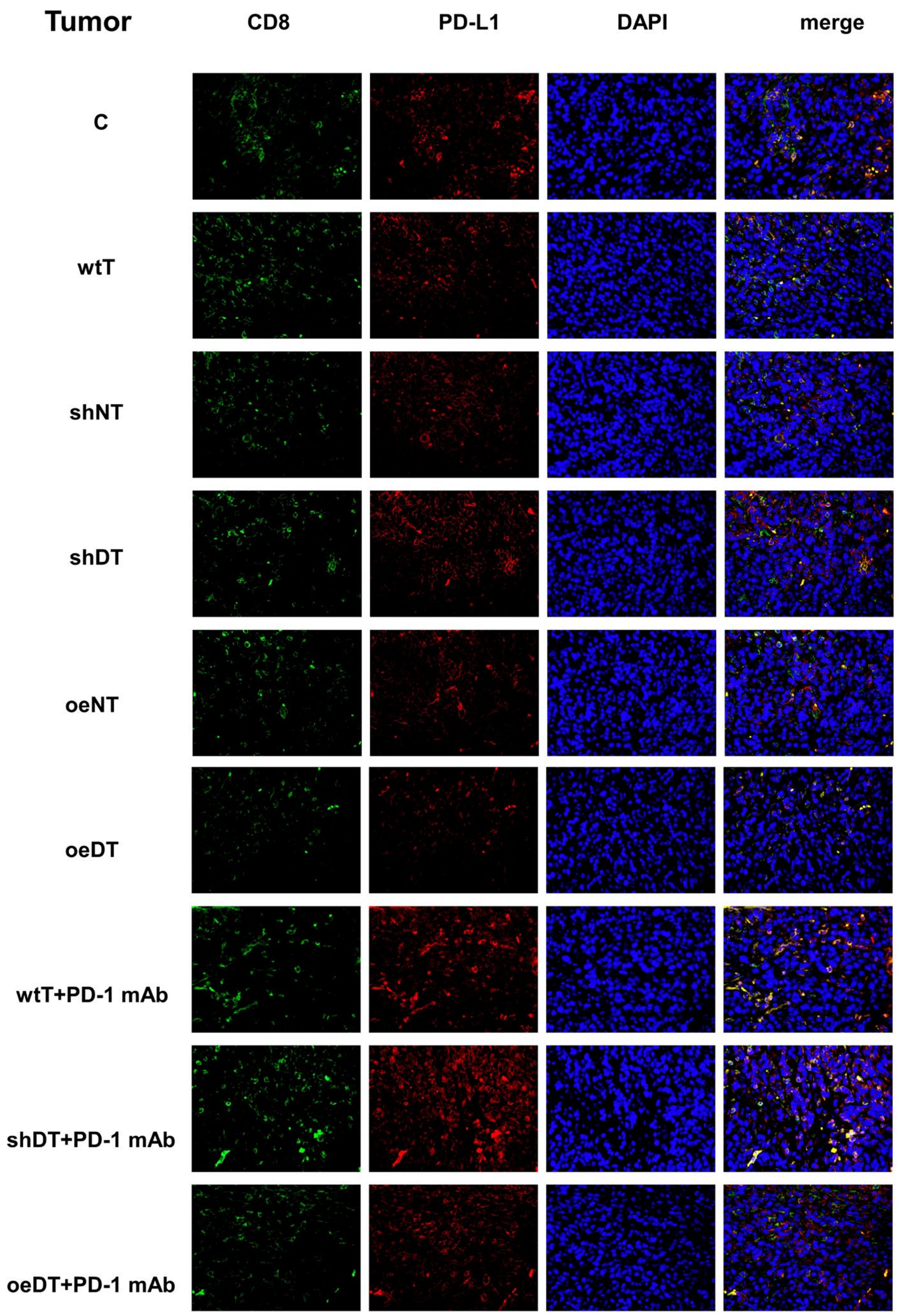

Fig.S7 Immunofluorescence images of the levels of $CD8^+$ T cells, $PDL1^+CD8^+$ double-positive T cells, and PD-L1 expression in tumor tissues of the different groups Abbreviations: C: control; wtT: wild-type $CD3^+$ T cells; shDT: $CD3^+$ T cells with Drp1 knockdown; oeDT: $CD3^+$ T cells with Drp1 overexpression; shNT and oeNT: negative control groups of shDT and oeDT, respectively; PD-1 mAb: programmed cell death protein 1 monoclonal antibody.

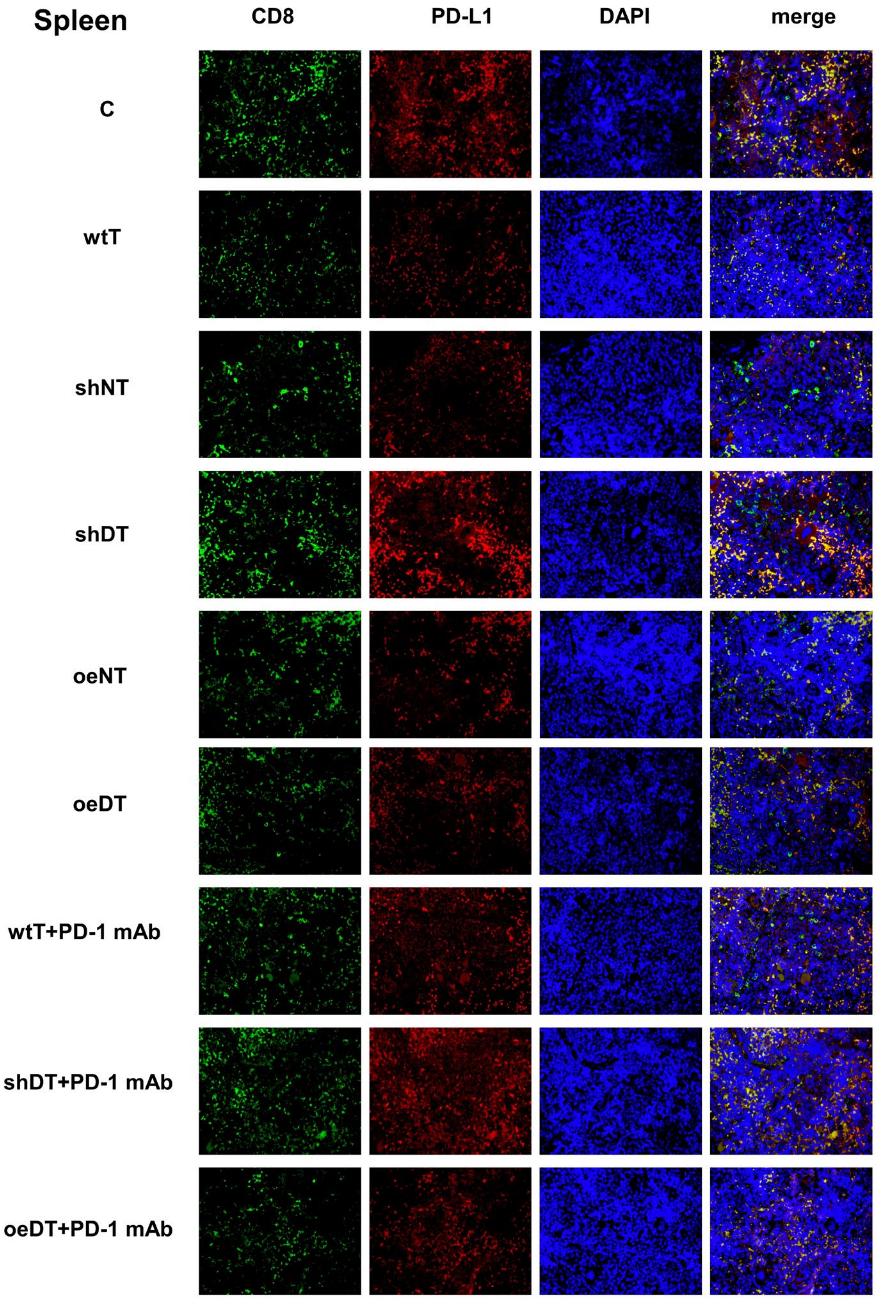

Fig.S8 Immunofluorescence images of the levels of $CD8^+$ T cells, $PDL1^+CD8^+$ double-positive T cells, and PD-L1 expression in the spleen of different groups

Abbreviations: C: control; wtT: wild-type $CD3^+$ T cells; shDT: $CD3^+$ T cells with Drp1 knockdown; oeDT: $CD3^+$ T cells with Drp1 overexpression; shNT and oeNT: negative control groups of shDT and oeDT, respectively; PD-1 mAb: programmed cell death protein 1 monoclonal antibody.

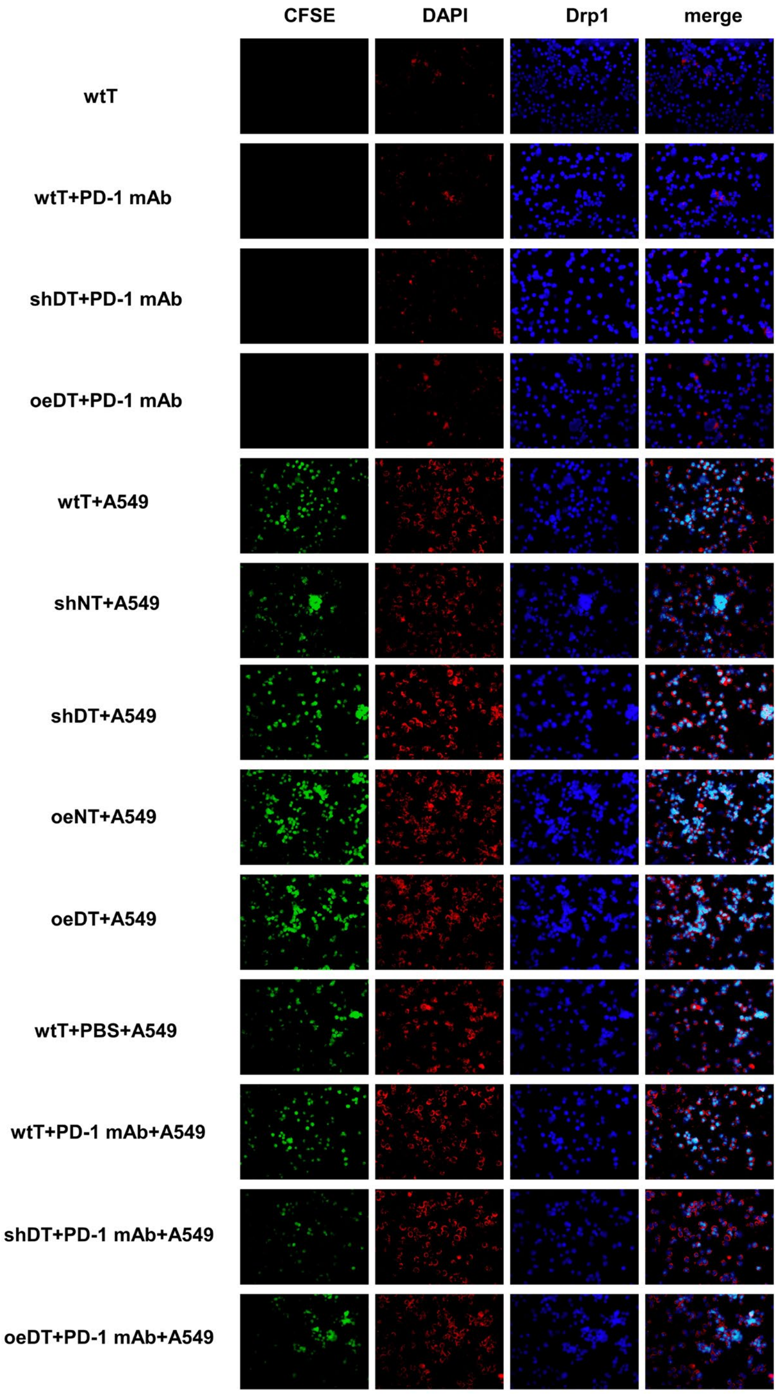

Fig.S9 Immunofluorescence images of Drp1 expression in T cells of the different co-culture groups

Abbreviations: wtT: wild-type $CD3^+$ T cells; shDT: $CD3^+$ T cells with Drp1 knockdown; oeDT: $CD3^+$ T cells with Drp1 overexpression; shNT and oeNT: negative control groups of shDT and oeDT, respectively; PD-1 mAb: programmed cell death protein 1 monoclonal antibody; PBS: phosphate-buffered saline.

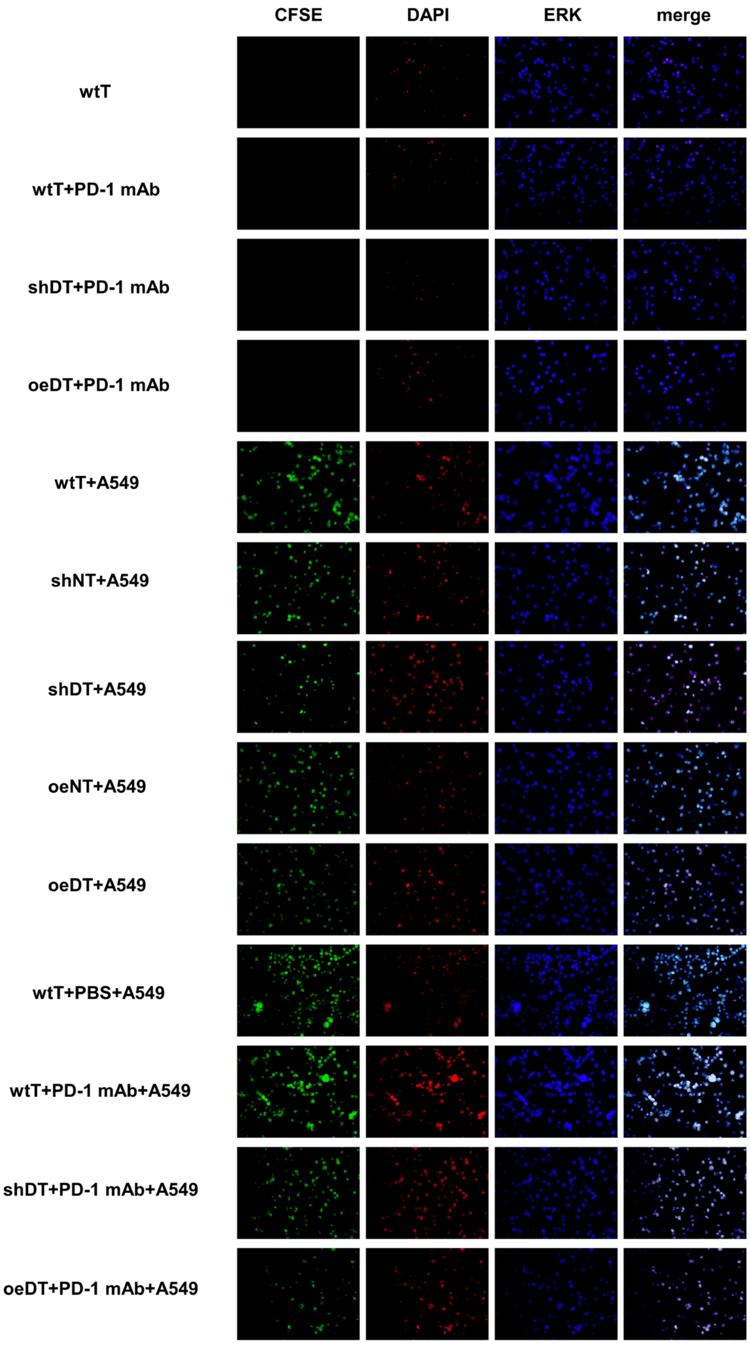

Fig.S10 Immunofluorescence images of ERK expression in T cells of the different co-culture groups

Abbreviations: wtT: wild-type $CD3^+$ T cells; shDT: $CD3^+$ T cells with Drp1 knockdown; oeDT: $CD3^+$ T cells with Drp1 overexpression; shNT and oeNT: negative control groups of shDT and oeDT, respectively; PD-1 mAb: programmed cell death protein 1 monoclonal antibody; PBS: phosphate-buffered saline.

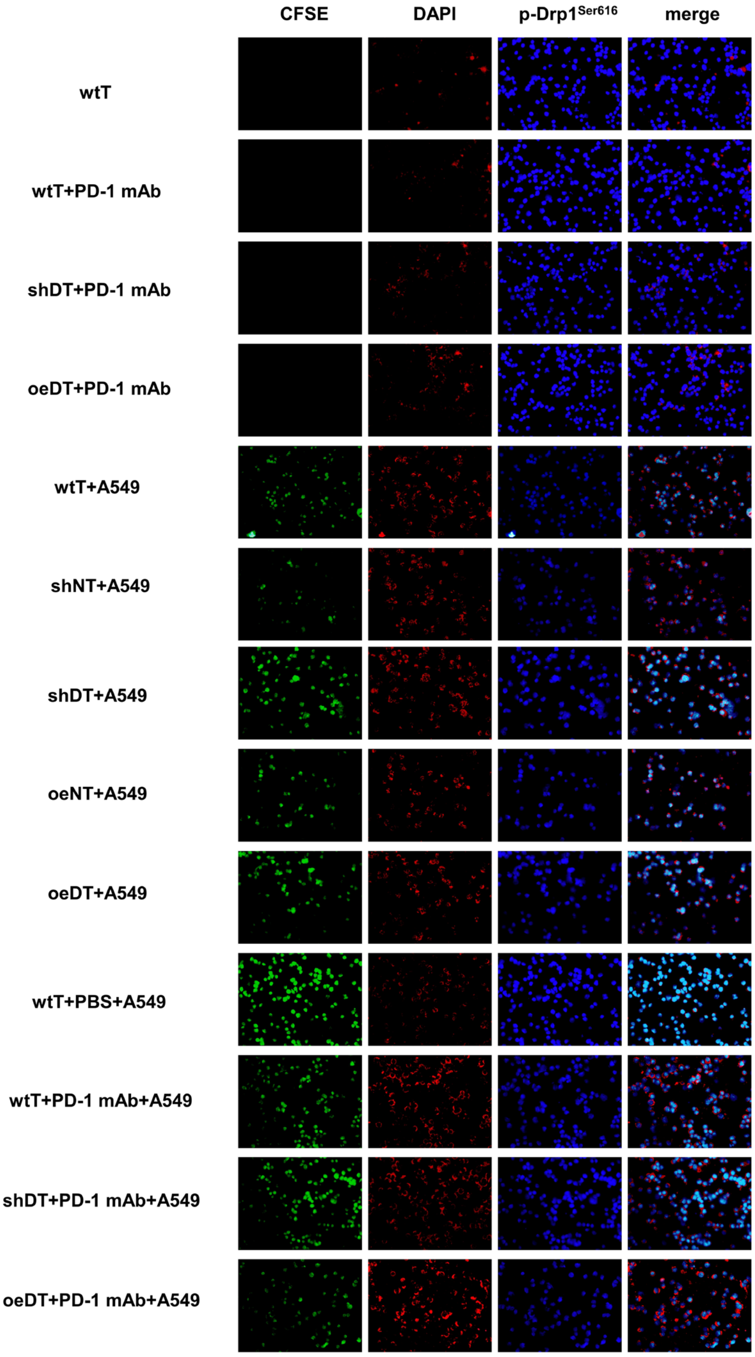

Fig.S11 Immunofluorescence images of p-Drp1^{Ser616} expression in the different co-culture groups

Abbreviations: wtT: wild-type CD3^{+} T cells; shDT: CD3^{+} T cells with Drp1 knockdown; oeDT: CD3^{+} T cells with Drp1 overexpression; shNT and oeNT: negative control groups of shDT and oeDT, respectively; PD-1 mAb: programmed cell death protein 1 monoclonal antibody; PBS: phosphate-buffered saline.

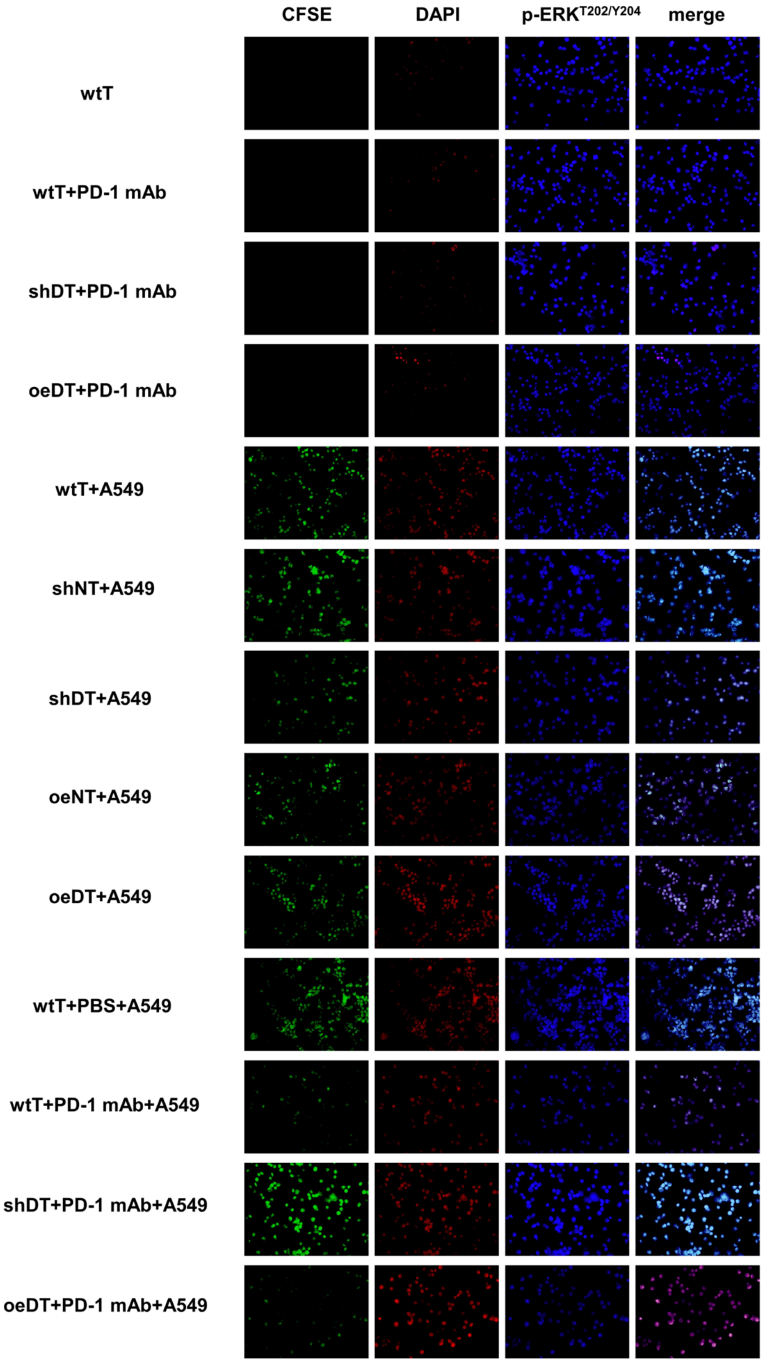

Fig.S12 Immunofluorescence images of p-ERK$^{T202/Y204}$ expression in the different co-culture groups

Abbreviations: wtT: wild-type CD3^{+} T cells; shDT: CD3^{+} T cells with Drp1 knockdown; oeDT: CD3^{+} T cells with Drp1 overexpression; shNT and oeNT: negative control groups of shDT and oeDT, respectively; PD-1 mAb: programmed cell death protein 1 monoclonal antibody; PBS: phosphate-buffered saline.
